# Validating a Termite-Inspired Construction Coordination Mechanism Using an Autonomous Robot

**DOI:** 10.3389/frobt.2021.645728

**Published:** 2021-04-21

**Authors:** Nicole E. Carey, Paul Bardunias, Radhika Nagpal, Justin Werfel

**Affiliations:** ^1^John A. Paulson School of Engineering and Applied Sciences, Harvard University, Cambridge, MA, United States; ^2^Wyss Institute for Biologically Inspired Engineering, Harvard University, Cambridge, MA, United States; ^3^Department of Biological Sciences, Florida Atlantic University, Boca Raton, FL, United States; ^4^Department of Civil and Environmental Engineering, South Dakota School of Mines, Rapid City, SD, United States

**Keywords:** biorobotics, humidity, stigmergy, collective construction, termite, template

## Abstract

Many species of termites build large, structurally complex mounds, and the mechanisms behind this coordinated construction have been a longstanding topic of investigation. Recent work has suggested that humidity may play a key role in the mound expansion of savannah-dwelling *Macrotermes* species: termites preferentially deposit soil on the mound surface at the boundary of the high-humidity region characteristic of the mound interior, implying a coordination mechanism through environmental feedback where addition of wet soil influences the humidity profile and vice versa. Here we test this potential mechanism physically using a robotic system. Local humidity measurements provide a cue for material deposition. As the analogue of the termite's deposition of wet soil and corresponding local increase in humidity, the robot drips water onto an absorbent substrate as it moves. Results show that the robot extends a semi-enclosed area outward when air is undisturbed, but closes it off when air is disturbed by an external fan, consistent with termite building activity in still vs. windy conditions. This result demonstrates an example of adaptive construction patterns arising from the proposed coordination mechanism, and supports the hypothesis that such a mechanism operates in termites.

## 1. Introduction

**Figure 1:**
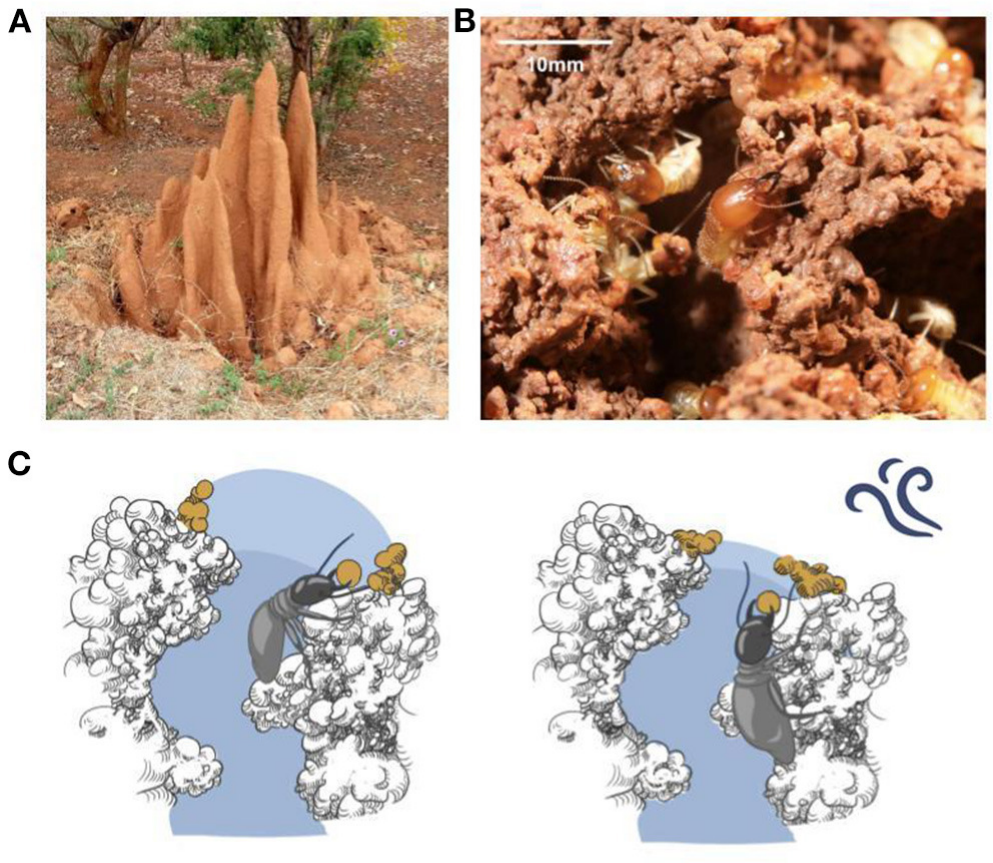
Mound-building termites and the humidity template hypothesis. **(A)** An *Odontotermes obesus* mound in India, ~1.2 m tall; mounds of other species can be much larger and have been reported at over 8 m (McFarlan and McWhirter, 1991). **(B)** Individual termites building at the end of a tunnel at the mound surface. **(C)** The hypothesis is based on agents depositing material at the edge of a region of elevated humidity (light blue shading), which extends a short way into the external environment when air is still, and terminates at the tunnel end when disturbed, leading to the tunnel being extended in the first case and closed off in the second, as described in Bardunias et al. (2020).

Mound-building termites of several different genera are known for their prowess in collective construction: colonies of millions of insects construct mounds that can be several meters tall, with elaborate outer features and complex networks of internal tunnels (Figures 1A,B; McFarlan and McWhirter, 1991; Turner, 2000; King et al., 2015). These examples from nature have long spurred interest in collective construction both from a scientific viewpoint, seeking to understand principles underlying the insects' activity (Grassé, 1959; Camazine et al., 2001), and from the engineering one, seeking to create artificial systems that operate under similar restrictions and with similar advantages (Werfel et al., 2014; Petersen et al., 2019). Mechanisms for coordinating the activity of independent agents acting with limited information are of interest to both communities. Understanding the principles behind the operation of such natural systems can provide a source of tools for designing artificial ones, as well as illuminating how these insect colonies function.

The classic explanation for how termites coordinate their building activity is based on a putative cement pheromone: a hypothetical chemical added to pellets of soil deposited by workers, which attracts other workers to the same site and triggers further deposition (Grassé, 1959; Bruinsma, 1979). However, a growing body of evidence has recently called this into question, indicating that such a pheromone may not be a primary cue used by the insects, or even that no such chemical (as traditionally construed) may exist (Fouquet et al., 2014; Petersen et al., 2015; Green et al., 2017). In its place, other cues and mechanisms that play a role in the coordination of termite building activity have been identified (Green et al., 2017; Calovi et al., 2019).

We and others recently described a novel mechanism based on sensitivity to air humidity (Bardunias et al., 2020), in which a zone of high humidity extends beyond the end of a tunnel into the outside world, and termites deposit wet soil at the edge of this zone (Figure 1C). The “bubble” of high humidity acts as a template for deposition. When the bubble is undisturbed, the deposition of additional wet soil moves the high-humidity zone outwards, creating a feedback loop allowing extension of the tunnel and expansion of the mound; when disturbed by external factors such as wind, the high-humidity zone ends closer to the mouth of the tunnel, and deposition at its edge leads to the tunnel being sealed off. This mechanism is consistent with observations on *Macrotermes michaelseni* mounds in Namibia (Bardunias et al., 2020).

**Figure 2:**
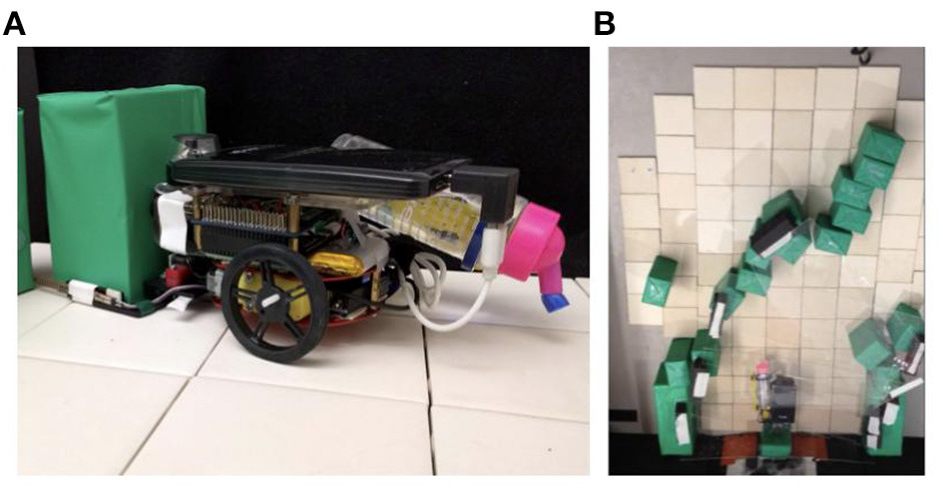
Robophysical model. **(A)** Robot agent analogous to a termite, with gripper for manipulating blocks at left of image and dripper for water deposition on the right. **(B)** Overhead view of the arena after the robot has placed several blocks.

While this humidity template mechanism is consistent with observed termite behavior, it is difficult to experimentally isolate humidity from other cues potentially available to termites (e.g., carbon dioxide level or air turbulence) and determine with certainty that their actions are solely due to humidity levels. To explore whether the humidity template mechanism on its own can produce the kinds of building patterns seen with the insects, in this work we test an artificial agent-based construction system in which humidity alone is the defining template characteristic. Simulations can indicate the feasibility of the basic mechanism (Supplementary Material), but simulations by necessity neglect factors that can prove to be of importance in the actual behavior of the simulated system (Zubair et al., 2011); using physical hardware ensures that an experiment correctly captures the physical features of the real world. In addition to their higher mechanistic fidelity, physical platforms can reveal interplays between agents and their environment not evident or easily reproduced in simulation (Harvey et al., 2008; Rubenstein et al., 2014; Wilson et al., 2018). More broadly, robots have frequently proven valuable as a testbed for hypotheses in studies of animal behaviors in past work (Webb, 2000, 2001; Aguilar et al., 2018; Digumarti et al., 2018). We therefore perform physical experiments using a purpose-built autonomous robot, which manipulates building blocks in an arena analogous to the conditions near the surface of a termite mound (Figure 2).

## 2. Materials and Methods

We constructed an autonomous mobile robot equipped with humidity sensors (Carey, 2019), and an arena representing a 2D analogue of the end of a termite mound tunnel where it opens to the outside environment (Figure 2). To model the water that a termite would add to the system via deposition of wet soil, we equipped the robot with a reservoir and dripper mechanism, and provided an absorbent substrate (unglazed ceramic tile, which when saturated evaporates at a rate of 0.75 g/h at 40% ambient humidity, comparable to the clay-heavy soil used by mound-building termites in northern Namibia at 0.6 g/h. These values were measured in laboratory conditions; details in Supplementary Material). The robot was primarily controlled using a Raspberry Pi, with supporting sensory information provided by an omnidirectional vision camera and two flexible bend sensors acting as pseudo-antennae. As the analogue for soil pellets, the robot manipulates blocks made of floral foam, wrapped in contact paper for high visual contrast, which are grasped between two forward prongs and secured or released via a motor-powered latch.

**Figure 3:**
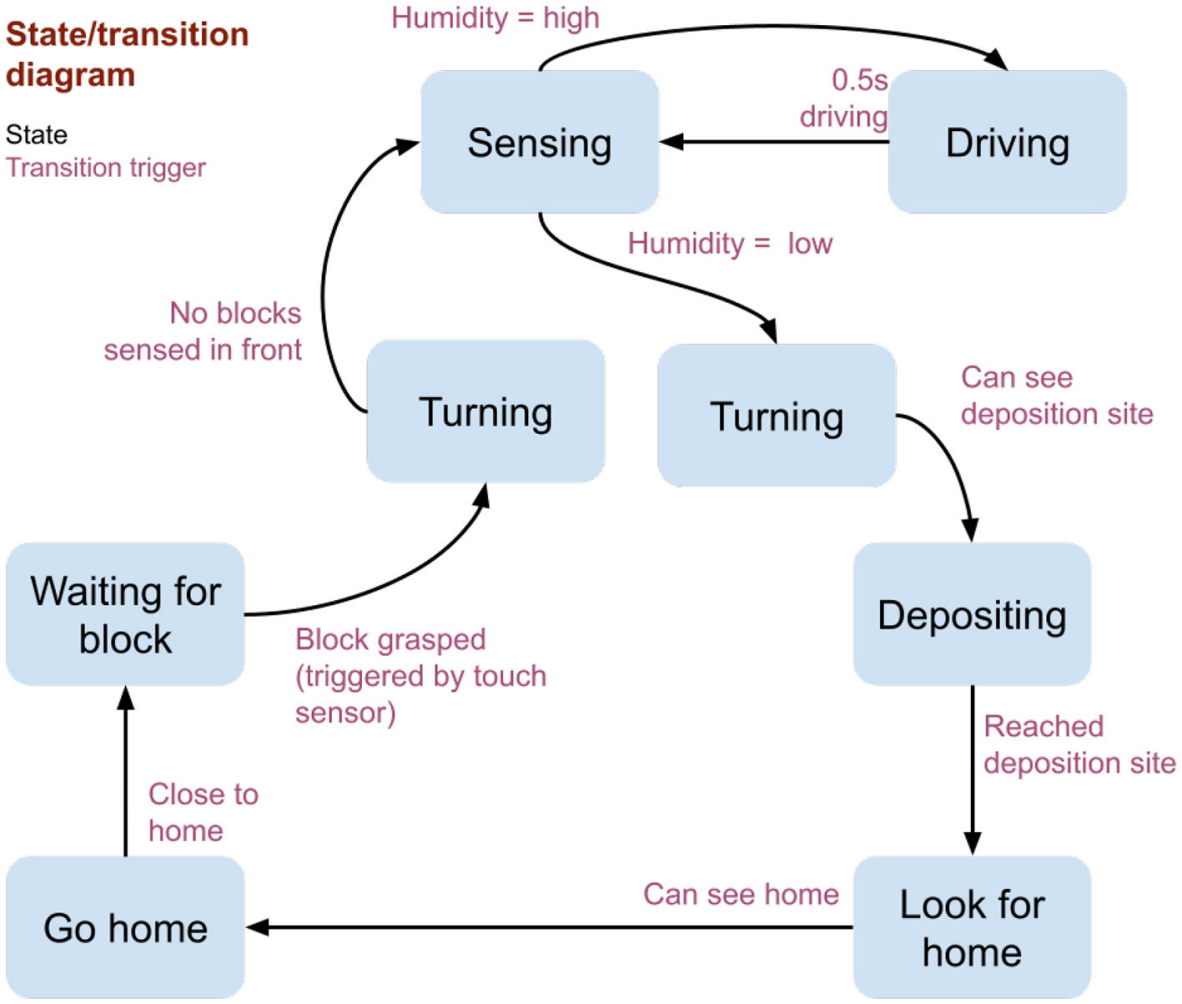
State and transition diagram used by the robot termite. Blue boxes indicate the robot states, and transition arrows between each state are labeled with the fulfillment conditions.

Initially, a pair of parallel walls provide a short tunnel; tiles between the walls are saturated, while those beyond the tunnel's end are left dry. A humidity bubble, like that observed during active building on the termite mounds (Bardunias et al., 2020), forms at the boundary (Supplementary Figure 1). The state and transition diagram governing the robot control is shown in Figure 3. During each experiment, the robot repeats the following loop: (1) Collect a block from the rear of the tunnel (manually provided through a opening normally kept covered). (2) Turn to face the front of the tunnel (detected via visual input). (3) Move forward in small steps, along the way sampling humidity at the front end of the robot 6 mm above the surface. (4) When relative humidity falls below a threshold of 75% RH, deposit the block adjacent to an existing wall or previously deposited block (analogous to a termite depositing pellets by attaching them to existing soil). (5) Return to the rear of the tunnel (using a visual landmark located near the block supply site to navigate).

To make a deposition, a termite must affix its load of saturated clay soil to existing material on the mound (Figure 1B). In our 2D analogue, the robot deposits its load adjacent to an existing wall or previously deposited block. The robot hence needs to accurately identify when it is sufficiently close to a wall to “attach” its load, before returning to the back wall of the arena. Termites are blind and navigate via smell, vibration, and touch; however, these modalities can be difficult to implement cleanly with current sensors, so deposition is accomplished using a combination of touch and the on-board omnidirectional camera (Supplementary Material).

As the robot moves, it passively drips water from its reservoir onto the substrate. To simulate the effect of the 3D nature of termite building in our 2D arena, a transparent acrylic lid covers the initial tunnel, and is manually extended after each deposition cycle to the furthest extent of any contiguous wall. The deposition loop repeats until either no space wider than 5 cm remains between deposited blocks, or 14 blocks have been placed.

We performed experiments under three conditions, analogous to those experienced by *M. michaelseni* workers in Namibia: (1) The external air is still, as is typical for termites building on the surface of their mound at night, or in protected areas on calm days. (2) A fan blows air across the arena outside the tunnel, corresponding to wind, typical especially during the day. (3) The robot's water reservoir is left empty, corresponding to conditions experienced by termites in the dry season.

These methods are described in detail in the Supplementary Material, along with supporting video of depositions under wet and dry still conditions.

## 3. Results

**Figure 4:**
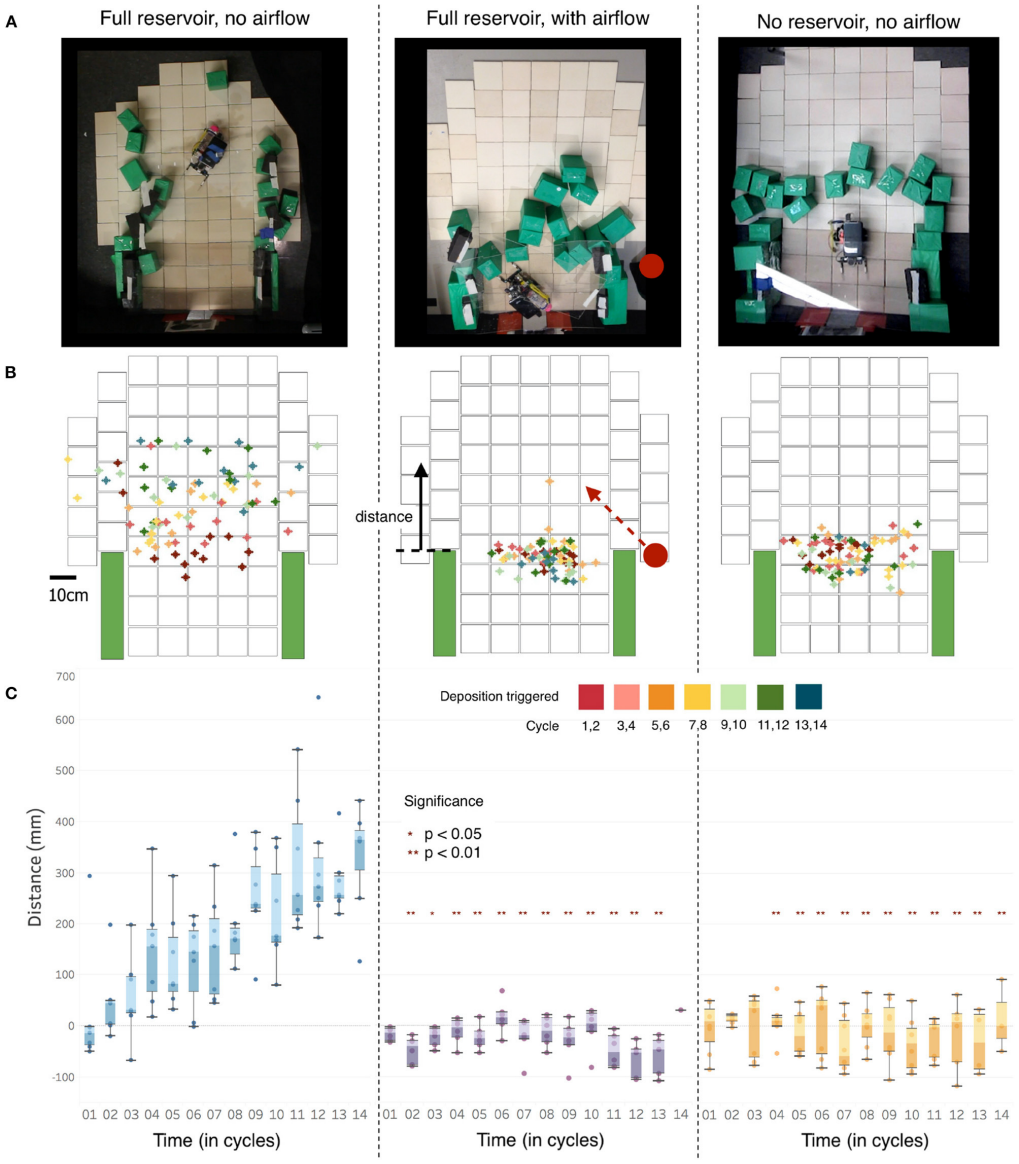
Results of robophysical experiments. **(A)** End state block placements for one example run of each of the three treatments [left: robot's water reservoir full, no external airflow; middle: external fan blowing across the arena from the right hand side (fan direction was alternated between experiments); right: no fan, water reservoir empty]. **(B)** Locations where measured humidity dropped below threshold (75% RH), triggering block deposition, superimposed for all trials for each condition. Cycle sequence is indicated by color of the location marker (inset), showing the change in trigger location over time. Fan position, shown by a red dot on the right in the middle panel, was alternated between trials. **(C)** Comparison of the distances of the block deposition trigger points from the initial wall length [see **(B)**, center] for the three experimental conditions. Significance calculated via 2-tailed independent sample *t*-test; a minimum of *n* = 6 trials was conducted for each experimental condition.

Figure 4 summarizes the results from the three experimental conditions described above. Row A shows a representative sample of the end-state block placement under each trial scenario. Row B shows the points at which the robot sensed the humidity had dropped below the given threshold value (75% RH), thus triggering a deposition, on a digitized map of the arena (determined by synchronizing the video feed from an overhead camera with the robot's own recording of the environment). The point where the robot passed the threshold for each block deposition cycle is marked with a cross; the color scale (inset) identifies the cycle sequence (up to a maximum of 14 blocks). Row C plots the vertical distance between the edge of the initial arena walls (marked in green in Row B) and the block deposition trigger points for all trials in the three experimental conditions.

In trials without external agitation of the air, the initial tunnel is extended outward as water deposition and new blocks trap humidity, allowing the robot to move increasingly further forward over time before deposition is triggered (Figure 4, left). In trials with a fan blowing air across the arena, the robot deposits blocks so as to close off the tunnel (Figure 4, center). Similarly, without additional water being added to the system, the zone of high humidity does not advance and the robot closes the tunnel (Figure 4, right). A two-tailed independent-sample *t*-test demonstrates a significant difference in deposition trigger location between the still high-humidity experiments vs. the disrupted and dry trials (Supplementary Material). The deposition distances of the blocks are not statistically different between the latter two experimental conditions, but a less orderly build structure in the fan case (Figure 4, Supplementary Figure 7) reflects the irregular evaporation pattern generated by the fan's disruption of the humidity bubble. End conditions for all trials in each experimental condition can be found in Supplementary Figures 8–10.

## 4. Discussion

The experiments above demonstrate that in the absence of disturbance to the humidity landscape, reinforcement of a humidity bubble through water associated with deposition can provide a feedback mechanism enabling mound expansion. Without such reinforcement, the expansion stops. In the robophysical experiments above, we observe that the humidity bubble formed in our arena is easily disrupted, with drier airflow reducing the humidity even close to the wet surface and behind the moderate protection provided by the block being carried. As a result of this disruption, the robot closes off the tunnel rather than continuing to extend it forward.

Theraulaz et al. (2003) make a distinction between template-driven mechanisms for building coordination, in which insects deposit material in locations based on physical heterogeneities in the environment, and stigmergy-based mechanisms, in which insects deposit material in locations based on the locations of previous depositions. While the adaptive construction mechanism described here is primarily template-driven (with agents depositing material based on the humidity gradient), there is a secondary stigmergic component in that new depositions must be physically contiguous with previously deposited material. The choice of deposition location dynamically modulates both the humidity template that defines the boundaries of the mound interior and the pattern of soil deposition, and is impacted by both.

The shape of the eventual construction thus depends on two factors. The first is the humidity template, which provides the cue directing agents to look for a place to deposit material; its shape is the result of moisture emanating (in the termite case) from the mound interior and freshly deposited material, or (in the robophysical experiments) from the wet tiles. The second is the evolving physical structure itself, which provides the substrate to which more material is attached; the locations of previous depositions shape the possibilities for further growth of the mound.

*Macrotermes michaelseni* building activity at the mound surface, expanding the mound, varies seasonally and with time of day. Almost all expansion occurs during or soon after the rainy season, when soil brought up by workers from below the nest is much wetter; in the dry season, when deep soils have much lower water content, little or no mound expansion occurs (Turner et al., 2006). During the rainy season, most surface building occurs at night, when the air is typically still and outside humidity is higher; in the daytime, when factors such as gusty winds and direct sunlight can disrupt the humidity bubble outside the protected confines of the mound, building activity at the surface is rarely seen. Building patterns in our simulation and robophysical experiments corresponded to these three conditions: In both types of experiments, the agent extends the tunnel when it transports water and the outside air is still, corresponding to termites building at night during the rainy season; it closes off the tunnel when the outside air is disturbed or it mobilizes no water, corresponding to termites building during the day or the dry season, respectively.

These results support the feasibility of the hypothesized mechanism for coordination of termite construction. While termites sense a wide range of environmental stimuli and are doubtless influenced by a variety of factors in different situations, these experiments indicate that humidity alone provides a sufficient cue for when to deposit material in order to produce such building patterns.

While the animal experiments that motivated this work were performed specifically with *M. michaelseni*, similar principles may operate with other insects. Many other termite species are observed to respond strongly to different humidity levels (Emerson, 1956; Yanagawa et al., 2010), including building behavior in particular (Howse, 1966). Other, more distantly related insects, such as leafcutter ants, also respond to dry airflow (Bollazzi and Roces, 2007), and could use similar principles in the construction of their nests. More broadly, interactions between dynamic environmental templates and stigmergic behaviors have been observed in other insects (Jost et al., 2007).

The challenges of swiftly navigating a largely symmetrical and homogeneous environment with limited visual fields became apparent through the experimental process, suggesting the advantages of incorporating biomimetic sensory modalities into robots that operate in visually constrained environments. Termites are blind and process the world largely through tactile and olfactory information. Coupling sensing of humidity (or analogous sensing of other chemicals or signals) with wide-field touch or force sensing may convey a richer, more salient landscape profile for such a construction algorithm. Nevertheless, the robot used here produced the observed building patterns despite its sensory limitations and imprecise navigation. Such robustness to unreliability of individual agents and actions is necessary to collaborative construction algorithms, for engineered systems no less than for social insect colonies, whose successful operation likewise cannot depend on unerring precision by each insect.

A template-based feedback process like the one considered here could potentially provide a novel mechanism to help coordinate environmentally-responsive building in collective robots. It requires no direct communication between agents, nor do agents respond directly to perceived configurations of material depositions, as they do in many insect-inspired models (Theraulaz and Bonabeau, 1995; Werfel et al., 2014). Rather, it is the indirect effect of the depositions on another relevant cue—in this case, local increases in air humidity—that prompts the local response by the agent, creating an environmentally mediated method of construction for scenarios where direct communication between agents may be unreliable or difficult. Philosophically, this approach hearkens back to early robotic investigations in collective construction, which saw a volatile template or environment as a key feature of such algorithms (Melhuish et al., 2006).

## Data Availability Statement

The original contributions presented in the study are included in the article/Supplementary Materials; further inquiries can be directed to the corresponding author/s.

## Author Contributions

NC, PB, JW, and RN: conceptualization. NC, PB, and JW: methodology. NC: robot hardware, software development, robot experiments, and visualization. JW: simulations and project administration. NC and JW: analysis and writing. RN and JW: supervision and funding. All authors contributed to the article and approved the submitted version.

## Conflict of Interest

The authors declare that the research was conducted in the absence of any commercial or financial relationships that could be construed as a potential conflict of interest.
