# Supplementary material for: Validating a Termite-Inspired Construction Coordination Mechanism Using an Autonomous Robot
Source: Front Robot AI. 2021 Apr 21;8:645728. doi: 10.3389/frobt.2021.645728 (PMC8098689; doi:10.3389/frobt.2021.645728)
Supplement: Supplementary file 1 [file Data_Sheet_1.PDF]

Supplementary Material

1 SUPPLEMENTARY METHODS

Arena and environment

The back of the arena, representing the mound interior, is enclosed on five sides by an absorbent substrate, a transparent acrylic lid, a backboard, and two fixed walls of building material (Fig. S1, right). The backboard has one small opening in the centre, through which blocks of building material may be inserted for retrieval by the robot. The chosen arena substrate was unglazed ceramic tile. To ensure the building material could be maneouvered easily by the light-weight mobile platform, the blocks used were cut from biodegradable floral foam, wrapped in contact paper for high visual contrast.

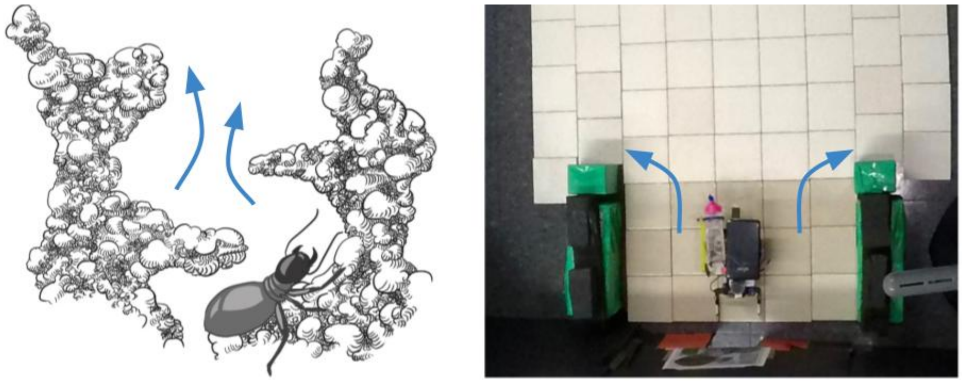

Figure S1. Diagram of a mound breach with high interior humidity (left), and the analogous experimental arena (right). Arrows represent the postulated direction of wet air diffusion.

We first tested whether the high-humidity bubble observed in situ on the mounds of *Macrotermes michaelseni* in Bardunias et al. (2020) also occurred at a larger scale using this substrate and block material. The rearmost three rows of unglazed ceramic tiles at the back of the arena were fully immersed in water for no less than five minutes before being placed into the arena. The remaining substrate was left dry (Fig. S1, right). This setup is the initial condition for each of the experimental trials reported. The three rows of wet tiles, fixed walls, and acrylic lid represent the end of an enclosed, open-ended tunnel on the mound; the fixed walls extend 50mm beyond the end of the last wet tiles in order to avoid biasing the potential formation of a humidity bubble beyond the tunnel's end.

We measured relative air humidity at the points shown in Fig. S2 using the SHT75 humidity sensors discussed below, by manually placing the robot at points in the arena and leaving it in place until sensor measurements stabilized (roughly 30s). This ensured the measurements were at the same height above the substrate as experienced by the robot, and hence representative of experimental conditions. The results showed a distinct bubble of high humidity extending beyond the saturated interior of the model tunnel. Based on these measurements, we chose 75% relative humidity as the threshold at which the robot switches to deposition behaviour: that value is reached at a distance 5–10cm out from the edge of the lid, or around half the robot's body length; analogously, on a termite mound under still conditions, the high-humidity bubble extends around half a worker's body length beyond the surface Bardunias et al. (2020).

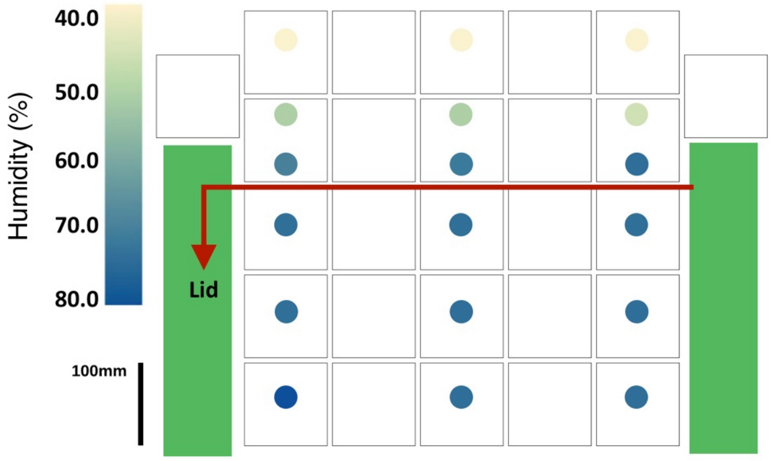

Figure S2. Humidity measurements of the experimental arena. The three tile layers closest to the base are pre-saturated and protected from the external environment by building block walls and a clear acrylic sheet. Other tiles are left dry and exposed. Humidity was measured using the experimental robot, by manually placing the robot at points around the arena and measuring the steady-state humidity.

Substrate evaporation

Unglazed ceramic tiles (100mm x 100mm) were obtained from a local vendor. To test evaporation rates, a single tile was weighed, and the equivalent weight of (acetone-washed) soil from a M. michaelseni mound was compressed into a disc of equal height and approximately similar surface area to the tile. Both tile and soil were then saturated with 25% water by weight (equivalent to the Attenberg plastic saturation state for this soil composition (Calovi et al. (2019))). The tile and soil were weighed every half hour for eight hours to obtain an average evaporation rate.

Robot

The robot design is shown in Fig. S3. An off-the-shelf Mini Round Robot from Adafruit provided the chassis and motors, while additional body components such as the water dripper mechanism were cut from sheet acrylic. The robot was controlled by a Raspberry Pi 3B with an onboard 5V 2A phone battery pack, plus additional PCB layers for sensing and motor control. To simplify manipulation, the robot acts only in two dimensions, gripping building blocks between two front-mounted shovel arms using a servo-powered wedge, and pushing them through the arena. The full implementation code can be found in Carey (2019).

Sensors

Humidity was measured by taking the average reading from three Sensirion SHT-75 sensors[1] mounted at the front of the robot, about 6mm above the tile surface (Fig. S3, left). Turbulent airflow from the robot's own motion can affect the SHT-75 response, so between each reading the robot comes to a complete halt and pauses for up to 30s to allow any flow underneath the chassis to settle. SpectraSymbol Flex Sensors were mounted at the front of the block manipulation forks to provide close-range contact information, while visual data was collected using an omnidirectional camera mounted at the front of the chassis (Fig. S3, centre).

[1] These have since been discontinued; the replacement Sensirion SHT-85 sensors have faster response time and are more consistent, and were used to create Video S1.

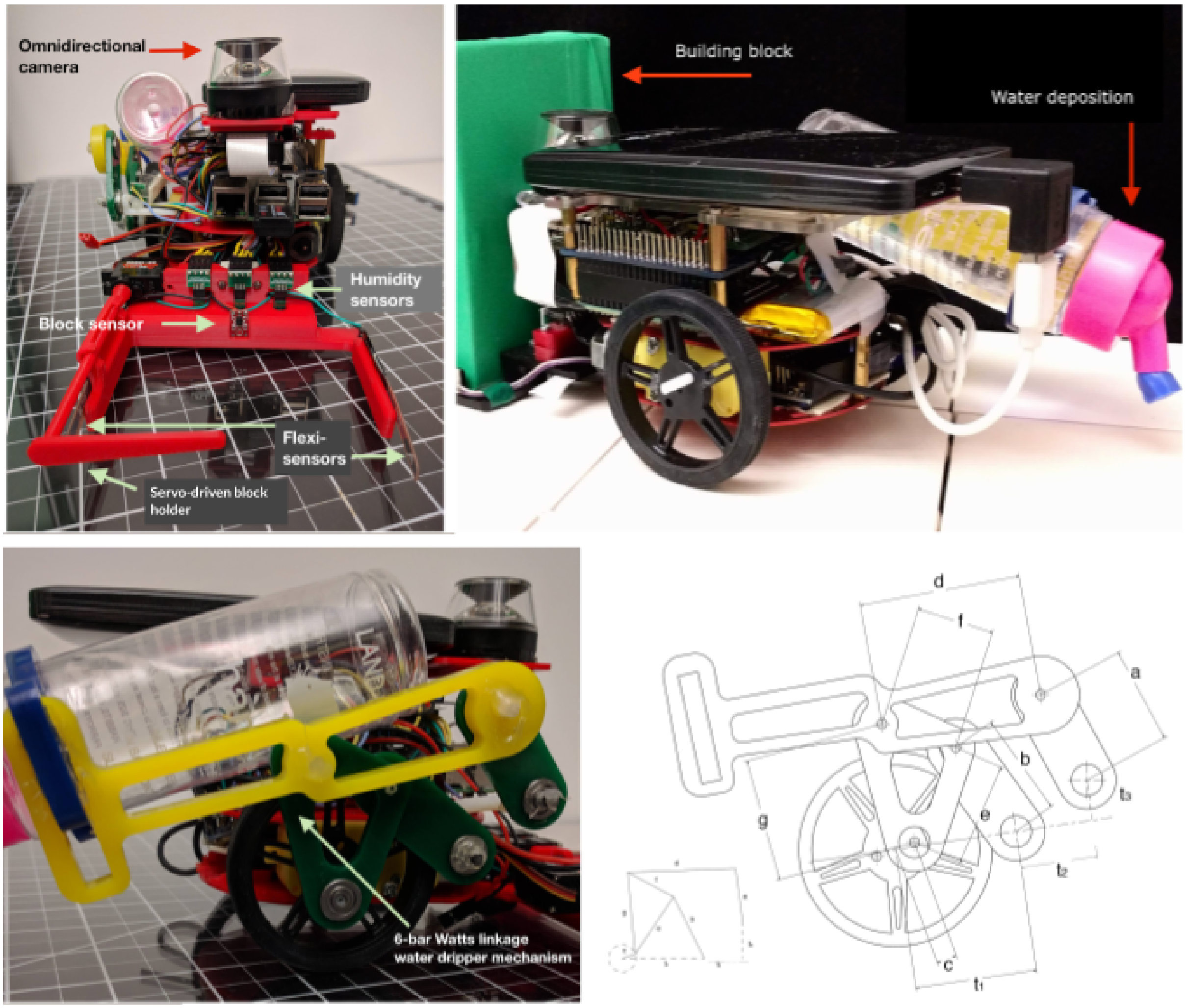

Figure S3. Mobile robot for humidity-responsive construction. Top left: front view, top right: robot in action. Humidity sensors extend below the robot's chassis, taking readings from 6mm above the tile substrate. The vision system is the highest point on the robot, maximising the field of view.Bottom left: side view of water deposition mechanism, bottom right: Watt's linkage with a = 30mm, b = 32mm, c = 6.25mm, d = 50 mm, e = 32mm, f = 24mm , g = 38mm , t_1 = 42 mm , t_2 = 23.25mm , $t3$ = 12mm

Vision System

Prior to experimentation, the chosen catadioptric camera system (a Raspberry Pi camera and an off-the-shelf hemispherical mirror for mobile phones) was calibrated to find its optical centre and enable the robot to discard non-salient information (the region of the camera's visual field extending beyond the mirror, and the region of the mirror reflecting the camera itself). The visual input was additionally filtered using HSV-thresholding to identify the (green, monochromatic) building material; and in order to enable more convenient expression of certain scene features, a pixel remapping function was generated to create an approximate 2D Cartesian representation of the scene, where the y-value of any pixel in the Cartesian remapping corresponds to its radial distance and the x-value corresponds to the angular position (Fig. S4). Note that this fast remapping is not a transformation to world coordinates, and cannot be used to reliably reconstruct egomotion, for example.

To provide additional robustness against potentially changing lighting conditions, the thresholding values were set automatically at the start of each experiment by calculating a hue-histogram from an initial snapshot and using a Gaussian filter around the relevant colour peaks.

Water deposition

The water reservoir carried by the robot was created from a small-animal automated dripper feed with a highly absorbent sponge in the nozzle (Fig. S3, top right). A six-bar Watts linkage (Fig. S3, bottom) enabled passive water deposition during the robot's travel, by allowing the saturated sponge to touch the arena floor at the low point of the linkage cycle.

Navigation

The agent's key decision in the humidity template hypothesis is when it stops travelling out away from the mound interior and begins instead to seek to deposit the material it holds; this decision is made based on its sensing of humidity as it travels. Other supporting tasks for the robot include: (1) ensuring the initial motion after picking up a block is towards the interior/exterior boundary, (2) locating a suitable deposition site, and (3) returning to the origin for more material. These tasks were accomplished using a combination of omnidirectional vision sensing and touch sensing from the forward-mounted flexion sensors. The key algorithms are described below:

Navigating towards the interior/exterior boundary

At the start of each deposition cycle, the robot is manually provided a block, whose presence it registers via a short-range reflection sensor mounted behind the arms. The robot seeks to identify a vector in the direction of the boundary by balancing the left and right walls visible in its visual field, excluding the central area occupied by the block. Once a suitable vector is identified, the robot thereafter ignores visual input and responds only to humidity cues.

Locating a deposition site

The robot repeatedly travels for 0.5s along its heading vector, pausing for 30s to allow air movements to settle, as recommended by the sensor manufacturer[2], and takes a humidity reading. When the humidity sensed by the robot drops below a pre-defined threshold (set to 75%RH for all experiments reported here), the robot transitions to a 'deposition' state, in which it uses its vision and touch sensors to find the edge of an existing wall or group of prior depositions, adjacent to which it could deposit the block it holds.

Field of view and camera placement are limited by the lidded arena, which places a physical cap on the robot height, and during deposition the building block being carried obstructs the frontal field of view (Fig. S4). Additionally, the lightweight blocks previously left in the arena may shift if the robot accidentally nudges them during a deposition process, meaning the shape of the space available to the robot is unpredictable and somewhat dynamic. The deposition algorithm therefore has to operate robustly even with incomplete information about the state of the arena. Consistent with a traditional desire for simplicity in swarm robotics and a parsimonious assumption about termite memory, we did not have the robot maintain any internal map of the arena, instead implementing a reactive algorithm using minimal levels of spatial reasoning or recollection.

To reduce bias in deposition direction, when the robot first measures the humidity as below the threshold, it makes a short blind turn for 0.1s, choosing left or right at random, It then calculates a probability metric which represents the likelihood of its current location being suitable for block deposition (details below). If

[2] in the case of the Sensiron SHT-85 sensors this settling period could be reduced to 15s

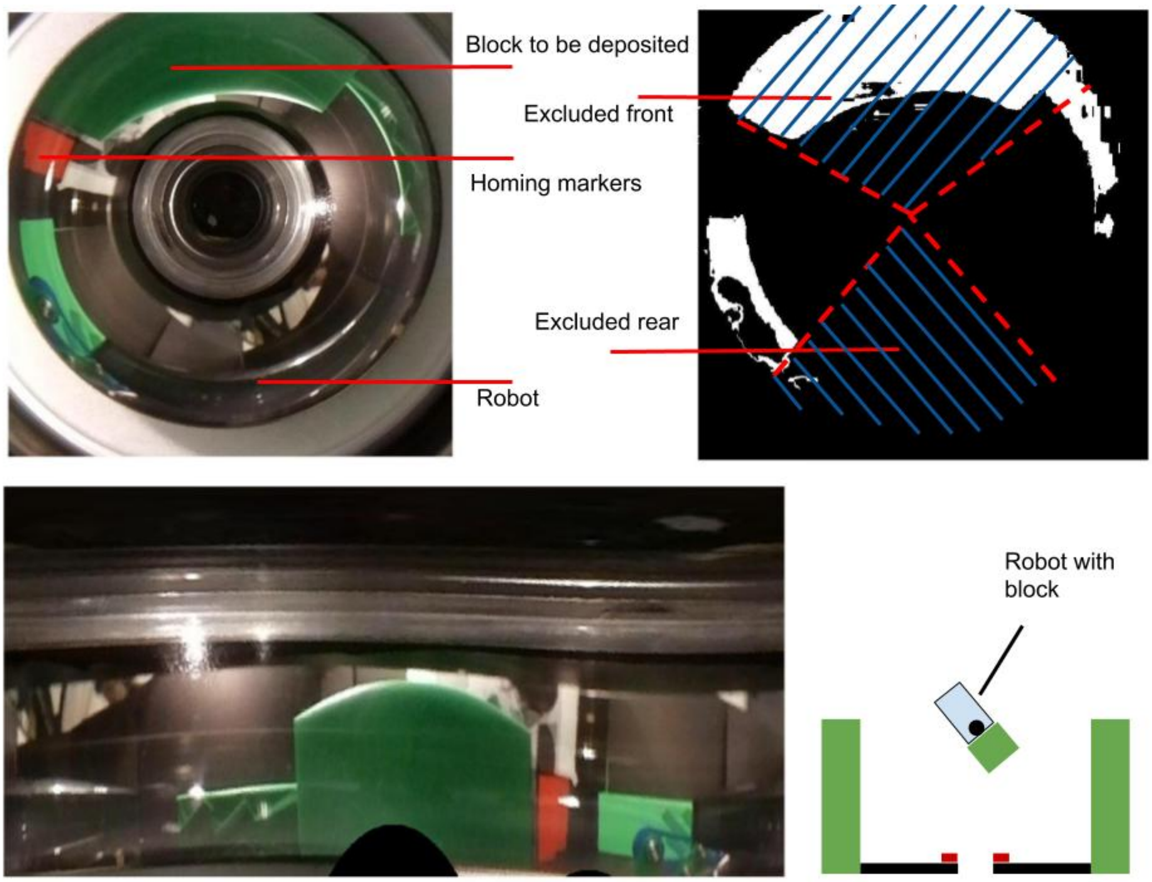

Figure S4. Top left: Unfiltered snapshot from the robot's omnidirectional camera. Top right: After applying an HSV filter with a green spectral peak, the forward and rear sections are excluded from analysis during deposition. Below left: An approximate pixel-based cartesian unwarping, with height/width corresponding to radial distance and angular position, respectively. Below right: Diagram of the physical arrangement corresponding to this snapshot.

this probability does not exceed a pre-defined threshold of 0.7 (see below), it either navigates towards the edge of any existing deposition, if visible, or continues to turn, if no existing deposition can be seen. To reduce processing overhead, the robot only updates its heading direction and localisation probabilities once every 0.5s.

Fig. S4 shows a typical omnidirectional scene during deposition. The diagram in the bottom right shows the robot position at the point the snapshot was taken. The front portion of the visual field is obscured by the block to be deposited; the rear is obscured by the robot itself. The usable visual field is therefore mainly lateral, so the robot cannot visually assess the area directly ahead in the direction of travel, and must infer information from the visual cues on either side and its (forward-positioned) flex sensors. (Note that we initially tried using these flex sensors ('whiskers') alone as location estimators, which proved unreliable, particularly as they flex naturally as the robot moves; however, when combined with visual cues, their input become a robust metric for wall proximity.)

Location cues: When the robot is sufficiently close to a wall to deposit, we expect at least one flex sensor to register contact via deflection. Visual localisation cues available include the presence and size of existing blocks or walls. 'Walls' (contiguous stretches of building material) were identified as connected binary

regions in the green-filtered visual scene, after eliminating the front central part of the visual field (since this would always be dominated by the block to be deposited). A minimum of one wall must be visible for a site to be suitable for deposition. Height/width ratio of the largest wall region (in pixels) characterised the robot's proximity to the wall, along with the flex sensor responses. As the centre of the field of view is obscured by the block being carried, the robot steers by trying to keep at a 30° relative bearing to the centre of mass of the largest visible wall region. This centre of mass is calculated using the unwrapped image, and its x-coordinate x_p used to determine the robot's target direction:

$$\theta_H = \frac{1}{c} x_p + 15. \tag{S1}$$

where c is the unwarp scaling factor. The target direction is updated every 100ms.

The suitability of a site for deposition is evaluated with the help of a binary feature vector **z**. The chosen set of localisation features constituting **z** was as follows (with corresponding binary thresholds):

$$\{\mathbf{z}\} = \begin{cases} n & \text{Number of wall areas identified} \geq 1 \\ \rho^L & \text{Height/width ratio of largest wall area} > \delta_1 \\ b^l & \text{Left whisker bend (as offset voltage reading)} > \epsilon_1 \\ b^r & \text{Right whisker bend (as offset voltage reading)} > \epsilon_2 \end{cases} \tag{S2}$$

For this vision system and flex sensors, the constants $\{\delta_1, \epsilon_1, \epsilon_2\}$ were set at $\{1.5, 2.0mV, 2.0mV\}$. Analysis of site suitability was accomplished using a Bayesian sensor fusion mechanism, adapting the method of Cummins and Newman (2008), where instead of estimating the likelihood of being in a specific pre-mapped location, we estimate the likelihood of being in a suitable deposition location. A Poisson distribution with a peak at 1 was used as the error model for the number of wall areas, while all pixel measurements used a Gaussian error model. Since the whiskers are loosely constrained in one direction by the block being deposited, their error model was based on a skewed gamma distribution.

The localisation algorithm is recursive, starting with an initial pre-estimate of site suitability (see below). As the robot moves, it incorporates new sensor readings and updates the probability that it has arrived at a suitable deposition locale based on this new information:

$$p(L_s|Z^k) = \frac{p(z_k|L_s, Z^{k-1})p(L_s|Z^{k-1})}{p(z_k|Z^{k-1})} \tag{S3}$$

where z_k is the current set of sensor observations (details can be found in Cummins and Newman (2008)). In this way, $p(L_s|Z^k)$ calculates the probability that the current site is suitable, based on the super-set of sensor observations Z up to time k (without explicitly storing previous observations). The initial probability of being in a suitable goal location is calculated as

$$p(L_s|\mathcal{Z}^0) = \beta \frac{1}{n} \tag{S4}$$

where n is the number of sensing steps between acquiring a block and humidity falling below threshold, and β is a scaling factor (set at 0.3 for the experiments in this work). To simplify the calculations, we assume the feature detector errors are independent of the actual location of the robot, and of each other (i.e., observations of one feature do not inform us about the presence/absence of others). When the cumulative probability exceeds a chosen threshold value ($p(L_s|Z^k) > 0.7$), the robot can infer that it has arrived at an

appropriate location for deposition. It then deposits the block by opening the wedge holding the block in place, moving slightly forward, then backing up while turning (in the same direction it first turned after measuring the humidity as below threshold; this turning while reversing reduces the disruption to other blocks and expedites finding the homing markers in the next step), until the shovel/whisker system is clear and unobstructed. Finally, it transitions to homing.

Homing

Two red markers were used to provide visually distinct features for the block retrieval site at the rear of the initial tunnel. Unlike deposition locales, which may take on a broad range of superficially dissimilar visual appearances, this homing target remains relatively visually static; hence we can use a more straightforward feature detection methodology to guide the robot back to an area where it can obtain a new block. The eight corners of the red homing targets served as a set of high contrast ordered features.

When computing odometry from a single omnicamera image, it is traditional to use ground-based features, which can be assumed to be at a known height from the camera (Scaramuzza and Siegwart (2008)). The placement of our vision system, and the relative symmetry of the arena, afforded us no such luxury—the ground plane is not visible to the camera except at some distance from the robot, and lowering the camera impedes deposition localisation. However, by taking an initial feature selection snapshot at a known distance from the homing goal, and knowing that all selected features are on the same vertical plane, we can use a modified Euclidean approximation to calculate the distance and direction between the robot's location and the desired 'home' position, as follows:

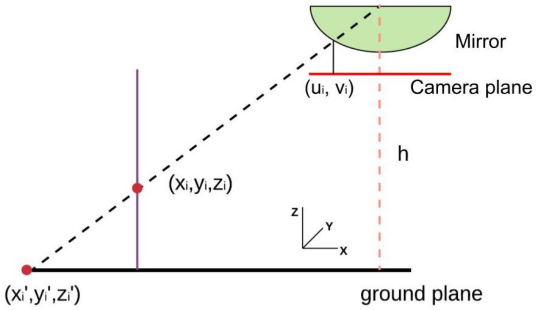

Figure S5. Line of projection of a feature in the mirror coordinate frame.

Let $\mathbf{u}_i = [u_i, v_i]'$ be the pixel positions of a feature $\mathbf{x}_i$ in the omnicam snapshot, and let ρ be the radial distance from the image centre $\rho_i = \sqrt{u_i^2 + v_i^2}$. The coordinates of the corresponding feature in the camera reference frame can be written $[x_i, y_i, z_i]'$, such that:

$$\mathbf{x}_i = \begin{bmatrix} x_i \\ y_i \\ z_i \end{bmatrix} = \lambda P \begin{bmatrix} u_i \\ v_i \\ f(\rho_i) \end{bmatrix} = \lambda P \mathbf{u}_i^+ \tag{S5}$$

where P is the perspective projection matrix of the camera system (Scaramuzza et al. (2006)). Projecting this point along the vector from the origin until it intercepts the ground, which is at a known (world) height

h (see Figure S5), we obtain the projected coordinates:

$$\mathbf{x}_i' = \lambda P \frac{h}{z_i} \mathbf{x}_i \tag{S6}$$

Note that for a catadioptric system, $P \approx I_2 - C$ where $C = [u_c, v_c]$ is the 2D centroid coordinates of the camera frame, in pixels. Hence, by taking an initial feature snapshot at a known distance x_S from the homing location, we can obtain an estimate for the initial scale factor $\lambda_S = x_S/(u_S - u_C)$ and from this calculate the height of each homing feature at the optimal stopping point, $z_S = \lambda_S f(\rho_S)$. This array of known heights provides a scaling factor that can be used to estimate distance from this location from a snapshot elsewhere in the arena.

When homing from an unknown position in the arena, the robot turns until it sees the red homing markers, then takes a snapshot from which it seeks to extract the features identified in the initial orientation phase. Let the features in the orientation snapshot be $\mathbf{X}_0$, and the corresponding set of features in the new homing snapshot be $\mathbf{X}_i$. Using the scaling factors from the initial snapshot to project all features from the vertical feature plane to the ground plane, as described above, we obtain the corresponding sets $\mathbf{X}_0', \mathbf{X}_i'$. Let the normal to the ground plane be $\mathbf{n}$, then we can write:

$$\mathbf{n}^T \mathbf{X}_0' = h \tag{S7}$$

and having thus remapped our vertical features into a set of ground-based features, we can proceed as for Scaramuzza and Siegwart (2008): for each view of the home location, we identify the pre-registered features $\mathbf{X}_i$, scale by their pre-calculated relative height to obtain a corresponding ground-based features set $\mathbf{X}_i'$, and find the Euclidean approximation to the rigid body transformation:

$$\mathbf{X}_i' = \mathbf{H}\mathbf{X}_0' = \left(\mathbf{R} + \frac{\mathbf{T}\mathbf{n}^T}{h}\right)\mathbf{X}_0' \tag{S8}$$

Solving this set of linear equations and using an SVD decomposition on the resulting rotation matrix $\mathbf{Q}$ to ensure an orthonormal solution, we obtain a vector between the current position and the home or goal position. The robot proceeds along this vector (with periodic re-sampling and estimation) until the distance between its position and the goal location drops below 0.1m.

Simulation

A simple lattice-based simulation can be used as a proof of principle to show the feasibility of the basic humidity template mechanism.

We created a 2D simulation representing building at a breach on the surface of a termite mound (Fig. S6). Each site of a discrete lattice has an associated humidity, and can either be occupied by building material or left empty. In the field, humidity inside a termite mound is high (relative humidity typically 96–98%) and outside is low (Bardunias et al. (2020)). The simulation represents the mound exterior as a line of occupied sites held at 0% humidity, in the middle of which is a breach represented by a set of three empty sites held at 100% humidity. Arid surrounding air is modeled by holding humidity at 0% for all sites outside a region that extends 5 sites in all directions beyond the extent of material added so far; when modeling wind across the surface of the mound, this region is shortened in the +y direction to 1 site beyond the extent of added material. Sites where material has been added are held at 10% humidity. Diffusion occurs by synchronously updating the humidity at all sites s_i such that a fraction D of the humidity H_i at s_i is transferred to each

neighboring empty site s_j, with a maximum humidity value of 100%:

$$H_i \leftarrow \min\left(H_i + D\sum_j (H_j - H_i)\,, 1\right)$$

This diffusion update is repeated 10^4 times between agent update steps to achieve equilibrium.

Agent behavior is as follows: A single agent starts at the leftmost site of the breach. At each agent update step, it moves to the next adjacent empty site following the wall formed by occupied sites. When it reaches a site where the humidity is below a threshold θ, that site becomes filled with material, and the agent is returned instantaneously to the rightmost site of the breach. From there it will follow the wall outward as before, returning to the left side of the breach after it reaches a site with humidity below θ, and so on alternating between left and right sides. As a result of the above rules, if the tunnel becomes completely closed off so that no passage exists between the breach and the outside world, no further deposition occurs.

In the simulations shown in Fig. S6, $D = 0.1, \theta = 0.75$.

Fig. S6 shows results of this simulation under three conditions. When the surrounding air is still, the humidity bubble extends beyond the breach or tunnel end, allowing the agent to move to the tunnel's rim before depositing, so that it builds the tunnel to extend forward beyond the breach indefinitely (Fig. S6A,B). When wind cuts off the bubble closer to the breach or tunnel end, the agent deposits material within the tunnel mouth and seals it off (Fig. S6C). If the environment is taken to be initially still and later windy, the agent builds the tunnel forward during the still phase, and closes it off when the windy phase begins (Fig. S6). These qualitative results are in accord with termite building (Bardunias et al. (2020)), and show that the humidity-template mechanism can produce such building patterns without the need for other environmental cues.

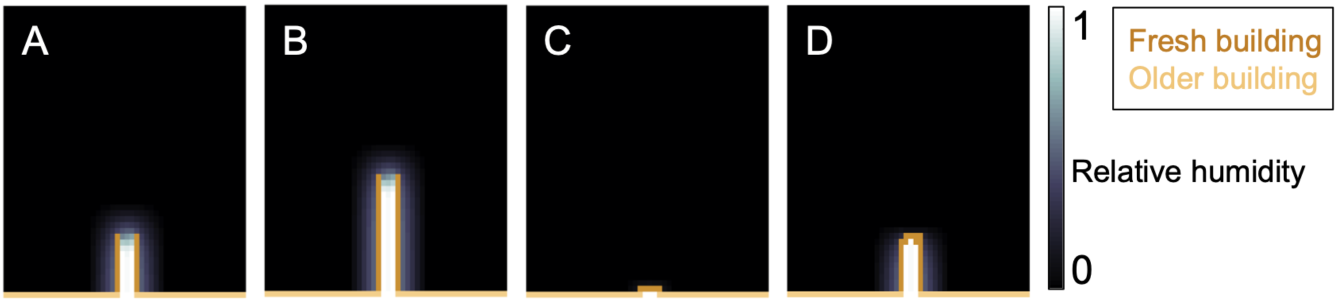

Figure S6. Simple simulation model. (A, B) With still air, an agent acting according to the postulated humidity-template process builds a tunnel that extends indefinitely from a breach. (A) shows the lattice after 20 depositions (110 agent update steps); (B) shows it after 40 depositions (420 agent update steps). Letting the simulation run still longer leads to the tunnel being extended still further, in the same way. (C) With wind disrupting the humidity profile beyond the breach, the agent seals it off. (D) If the air is still at first and later becomes windy, the agent extends the tunnel during the former period (here lasting for 20 depositions) and then closes it off when the latter begins. Light brown shows old building (the original mound surface, with a breach in the middle), darker brown shows fresh building (depositions placed by the agent during the run), grayscale shows relative humidity.

2 SUPPLEMENTARY DATA

Includes: Supplementary videos S1, S2, Supplementary figures S7-S10, Raw data files (pdf, csv)

Experimental conditions

Experiments were conducted indoors in an internal windowless room in Cambridge, MA (USA), in two batches - one in early spring (March-April) and one in late Autumn (Nov-Dec). Temperature in the room was held at 21C via the environmental heating and cooling system, ambient humidity was not directly controlled but ranged between 30%RH and 42%RH depending on external weather conditions.

Video analysis

For post-hoc analysis of the robot's behaviour, position data during experiments was extracted from an overhead digital video (Supplementary Video S1, S2), and synchronized with the robot's own recordings of humidity levels and its internal state. (This position data is calculated offline and is not available to the robot.) This information was used to reconstruct the point at which the humidity recorded by the robot dropped below threshold and it moved into seeking a deposition location, along with the final placement of the deposited blocks.

The supplementary videos S1, S2 show a dry (empty water reservoir) and wet (full water reservoir) experimental run, respectively, without external perturbation of the air.

End-state conditions

Figure S7 shows a histogram of the (discretized) end-state block position relative to the initial breach threshold. These distances can be compared using Shannon's discrete entropy metric (Martin and England (2011)), we find that the block placement in dry conditions is significantly less disordered than when the humidity envelope is disrupted by a fan..

Figures S8 - S10 show the end-state conditions for all trials, with the final block placement. The top half of each figure shows a snapshot from the overhead camera used to record the experiments, the bottom shows a digitized image of the same scene with the blocks labelled in order of dpeposition. Figure S8 shows the final states for experiments with a full water reservoir and no deliberate disturbance of the air in the arena. Figure S9 shows the final states with a full water reservoir and a fan placed such that air flow was induced diagonally across the arena (fan location is indicated by a red dot, with wind direction indicated by an arrow). Figure S10 shows the final states for still conditions but with an empty water reservoir.

REFERENCES

Bardunias, P. M., Calovi, D. S., Carey, N., Soar, R., Turner, J. S., Nagpal, R., et al. (2020). The extension of internal humidity levels beyond the soil surface facilitates mound expansion in Macrotermes. *Proceedings of the Royal Society B* 287, 20200894

Calovi, D. S., Bardunias, P., Carey, N., Turner, J. S., Nagpal, R., and Werfel, J. (2019). Surface curvature guides early construction activity in mound-building termites. *Philosophical Transactions of the Royal Society B* 374, 20180374

[Dataset] Carey, N. (2019). A Python implementation for a humidity sensing robot with omnidirectional vision. `https://github.com/niccarey/Leaky`

Cummins, M. and Newman, P. (2008). FAB-MAP: Probabilistic localization and mapping in the space of appearance. *The International Journal of Robotics Research* 27, 647–665

Martin, N. F. and England, J. W. (2011). *Mathematical theory of entropy*. 12 (Cambridge university press)

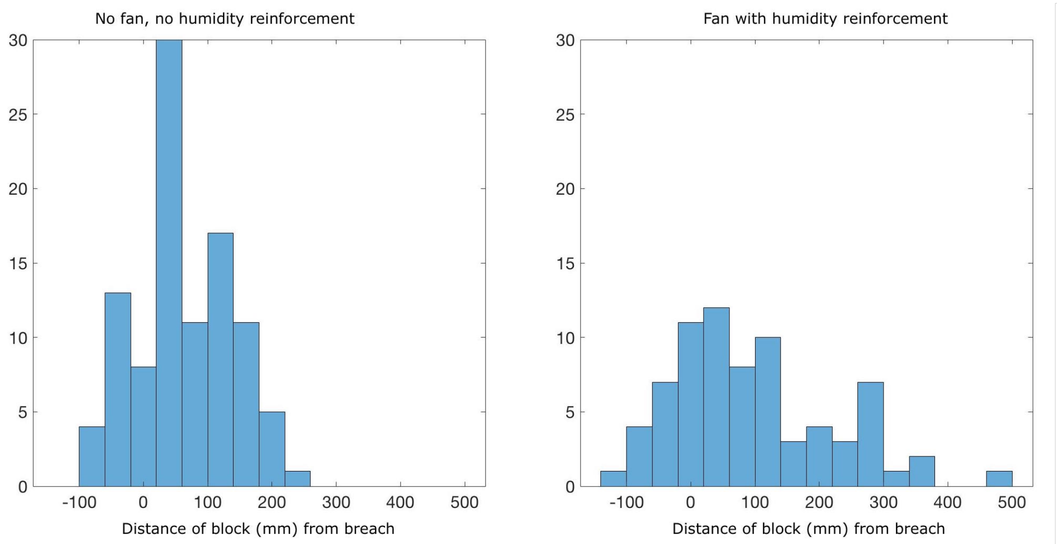

Figure S7. Histograms of block position in the arena relative to the initial breach or opening, for six trials of dry experiments (left) and wet experiments with fan (right). The corresponding entropy measures of the block distribution for these two conditions are $H_D = 1.94$, $H_F = 2.38$, reflecting the greater disorder in the fan-disrupted condition. Bin size is 40mm.

Scaramuzza, D., Martinelli, A., and Siegwart, R. (2006). A flexible technique for accurate omnidirectional camera calibration and structure from motion. In *Fourth IEEE International Conference on Computer Vision Systems (ICVS'06)* (IEEE), 45–45

Scaramuzza, D. and Siegwart, R. (2008). Appearance-guided monocular omnidirectional visual odometry for outdoor ground vehicles. *IEEE Transactions on Robotics* 24, 1015–1026

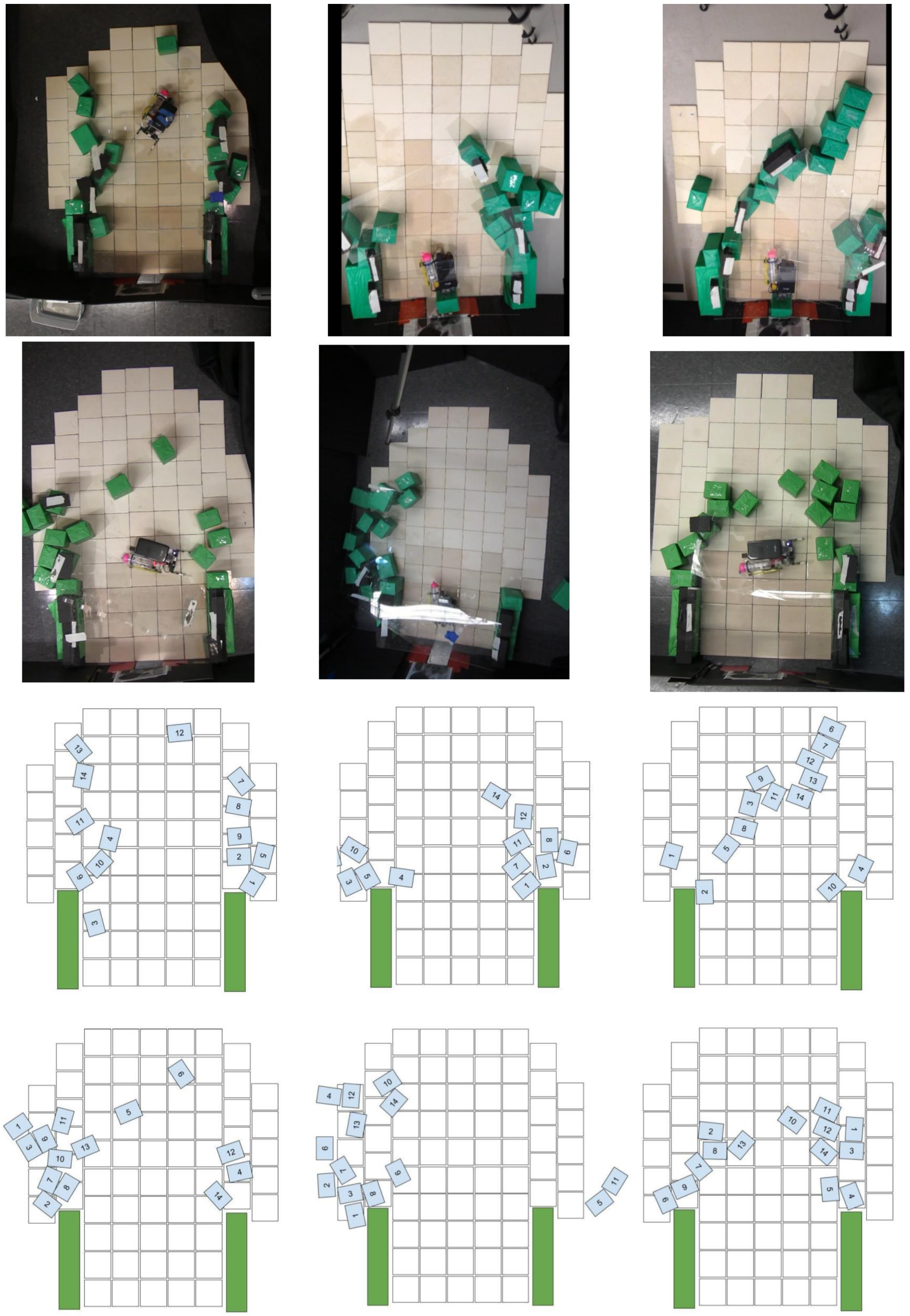

Figure S8. Final states of the robophysical experiments under still conditions with full water reservoir. Top: Video frames of block positions. Bottom: digitised reconstruction of block positions.

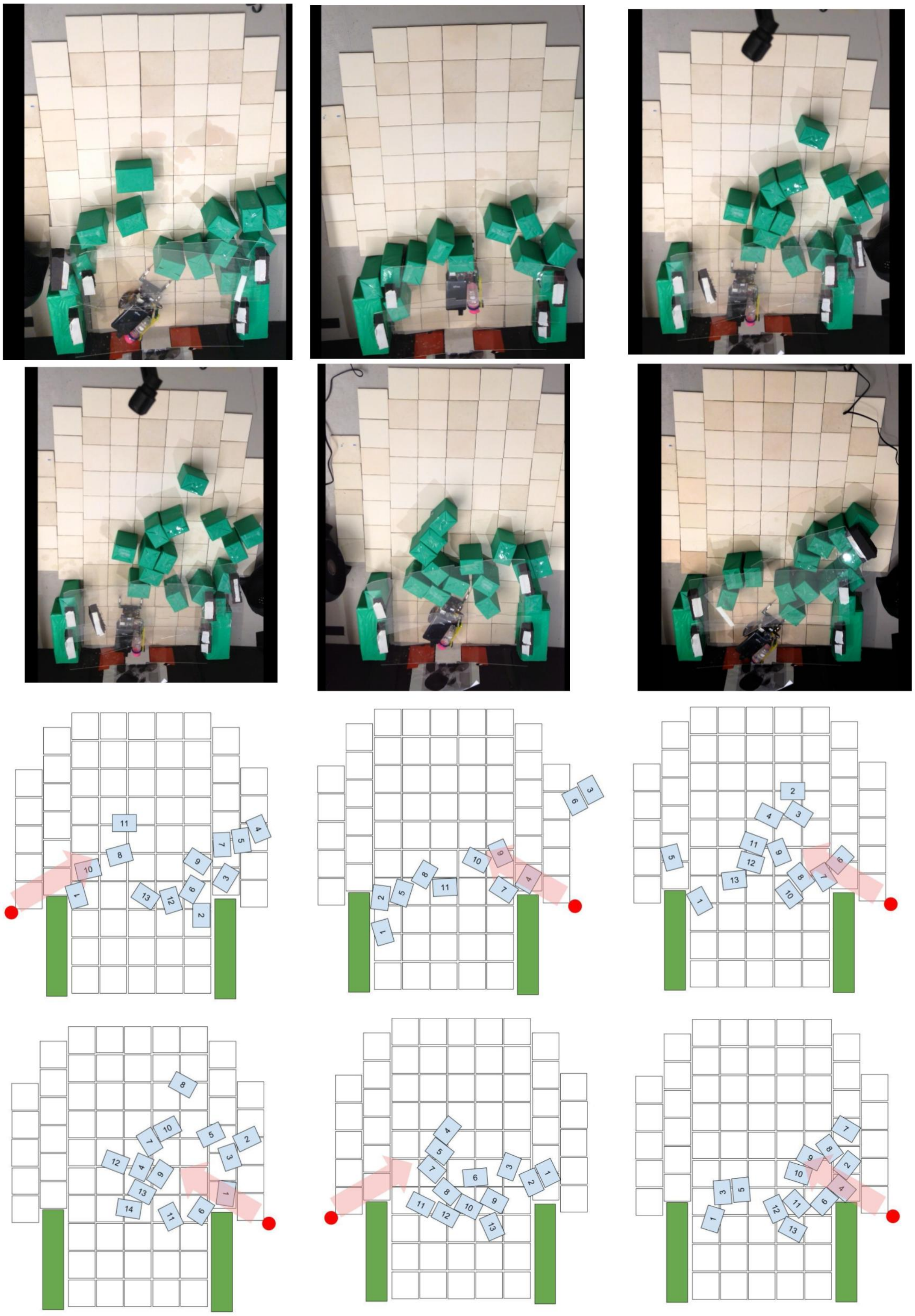

Figure S9. Final states of the robophysical experiments with disruptive wind and full water reservoir. Top: Video frames of block positions. Bottom: digitised reconstruction of block positions. Fan position is shown by a red dot, direction of wind disruption is given by the red arrow.

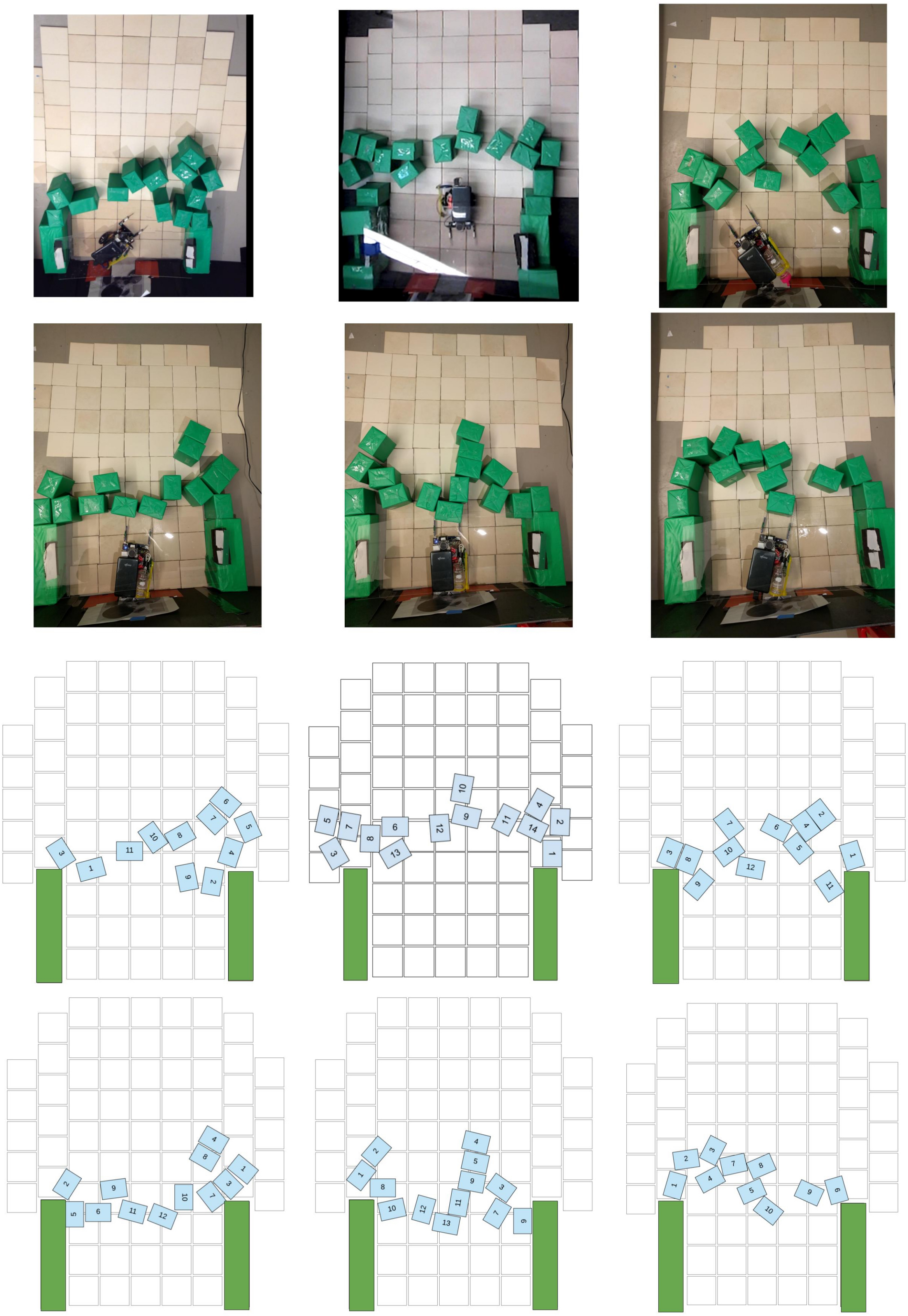

Figure S10. Final states of the robophysical experiments under still conditions, with an empty water reservoir (no humidity reinforcement over time). Top: Video frames of block positions. Bottom: digitised reconstruction of block positions.
