# Supplementary material for: Validating a Termite-Inspired Construction Coordination Mechanism Using an Autonomous Robot
Source: Front Robot AI. 2021 Apr 21;8:645728. doi: 10.3389/frobt.2021.645728 (PMC8098689; doi:10.3389/frobt.2021.645728)
Supplement: Supplementary file 2 [file Data_Sheet_2.PDF]

Stat Tests	B01	B02	B03	B04	B05	B06	B07	B08	B09	B10	B11	B12	B13	B14
**Dry**														
Mean turning distance (mm)	295.40	12.87	3.31	9.19	-13.97	8.09	-37.50	0.74	-12.50	-36.03	-26.05	-14.71	-31.62	13.73
Mean deposition distance (mm)	-8.82	293.12	276.12	243.93	221.75	248.67	222.36	198.12	158.73	147.89	142.11	117.04	138.15	172.78
Standard Deviation (turning)	58.82	8.46						40.10	58.33	50.38	38.74	66.50	68.49	71.58
**Fan**														
Mean turning distance (mm)	-16.67	-47.25	-22.06	-10.29	-22.55	16.67	-24.02	-11.76	-29.41	-5.39	-48.53	-67.06	-59.41	
Mean deposition distance (mm)	268.23	271.36	347.74	282.49	281.57	278.29	270.36	217.79	195.25	161.17	160.26	109.95	96.41	
Standard Deviation (turning)	12.57	25.95				40.36		28.58	40.97	49.35	32.37	39.97	40.13	

	B01	B02	B03	B04	B05	B06	B07	B08	B09	B10	B11	B12	B13	B14
**No fan**														
Mean turning distance (mm)	14.15	49.10	58.88	149.16	114.16	126.23	122.25	195.21	244.98	233.14	256.27	283.90	283.38	315.08
Mean deposition distance (mm)	401.63	347.29	356.92	344.12	397.53	469.93	446.13	336.05	389.95	420.53	411.71	455.30	465.26	407.24
Standard Deviation (turning)	113.76	71.30				84.66		88.64	98.631415[illegible]	160.37	124.64	163.33	68.05	101.67

2-tailed T (dry vs no fan)														
Turning Distance	0.70718	0.16773	0.08056	0.00315	0.00553	0.00489	0.00075	0.00008	0.00002	0.00081	0.00008	0.00042	0.00015	0.00239
Deposition Distance	0.01890	0.14238	0.02522	0.03721	0.00231	0.00030	0.00017	0.00085	0.00013	0.00022	0.00011	0.00013	0.00006	0.01369

2-tailed T (fan vs no fan)														
Turning Distance	0.44311	0.00359	0.00483	0.00139	0.00365	0.00476	0.00142	0.00006	0.00001	0.00187	0.00005	0.00014	0.00000	n/a
Deposition Distance	0.00665	0.11160	0.86894	0.42954	0.11377	0.04107	0.00574	0.14121	0.00071	0.00120	0.00029	0.00013	0.00002	n/a

Dry Runs	Turning distance from base (mm)	Turning height from wall (mm)	Block Height from wall (mm)	Block Dist from centre (mm)
Mar 2 Trial 1	400.00	47.06	100.00	379.76
Mar 2 Trial 2	347.06	-5.88	41.18	280.45
Mar 2 Trial 3	370.59	17.65	88.24	325.66
Mar 2 Trial 4	352.94	0.00	76.47	311.10
Mar 5 Trial 1	370.59	17.65	47.06	293.06
Mar 5 Trial 2	297.06	-55.88	23.53	295.58
Apr 5 Trial 2	267.65	-85.29	-5.88	170.59
Aug 6 Trial 1	402.94	50.00	20.59	307.03

No Fan Runs	NF Turning Distance from base (mm)	NF Turning height from wall (mm)	NF Block distance from centre (mm)	NF Block height from wall (mm)
Mar28 Trial 1	423.71	70.77	355.44	66.15
Mar28 Trial 2	302.94	-50.00	240.00	0.00
Apr4 Trial2	350.00	-2.94	383.85	120.00
Aug 8 Trial1	647.06	294.12	402.84	-32.31
Nov 17 Trial 1	339.1	-13.84	379.26	24.62
Nov 17 Trial 3	305.25	-47.69	491.49	192.31
Nov 18 Trial 1	265.25	-87.69	325.27	-230.77
Dec 6 Trial 1	319.1	-33.84	595.56	313.85
Dec 17 Trial 1	351.4	-1.54	440.97	298.46

Fan Runs	Fan Turning distance from base (mm)	Fan Turning distance from wall (mm)	Fan Block distance from centre (mm)	Fan Block height (mm)
Mar 29	350.00	-2.94	241.54	0.00
Mar 30	347.06	-5.88	275.22	-152.31
Apr 2	323.53	-29.41	227.20	-43.08
Apr 3	320.59	-32.35	333.12	50.77
Apr 4	344.12	-8.82	337.66	103.08
Apr 5	332.35	-20.59	194.66	-66.15

Dry Runs	Turning distance from base (mm)	Turning height from wall (mm)	Block Height from wall (mm)	Block Dist from centre (mm)
Mar 2 Trial 1	364.71	11.76	70.59	326.72
Mar 2 Trial 2	358.82	5.88	164.71	249.71
Mar 2 Trial 3	376.47	23.53	41.18	263.07
Mar 2 Trial 4	370.59	17.65	147.06	295.58
Mar 5 Trial 1	364.71	11.76	129.41	286.85
Mar 5 Trial 2	350.00	-2.94	55.88	372.80
Apr 5 Trial 2	367.65	14.71	-44.12	209.40
Aug 6 Trial 1	373.53	20.59	117.65	340.82

No Fan Runs	NF Turning Distance from base (mm)	NF Turning height from wall (mm)	NF Block distance from centre (mm)	NF Block height from wall (mm)
Mar28 Trial 1	437.56	84.62	234.54	32.31
Mar28 Trial 2	402.94	50.00	317.63	84.62
Apr4 Trial2	400.00	47.06	240.60	-16.92
Aug 8 Trial1	550.00	197.06	525.08	83.08
Nov 17 Trial 1	352.94	0.00	348.60	123.08
Nov 17 Trial 3	456.02	103.08	316.09	-209.23
Nov 18 Trial 1	328.32	-24.62	380.73	-327.69
Dec 6 Trial 1	332.94	-20.00	400.50	20.00
Dec 17 Trial 1	357.61	4.67	361.85	290.77

Fan Runs	Fan Turning distance from base (mm)	Fan Turning distance from wall (mm)	Fan Block distance from centre (mm)	Fan Block height (mm)
Mar 29	302.94	-50.00	234.98	-73.85
Mar 30	323.53	-29.41	236.51	-23.08
Apr 2	322.35	-30.59	384.83	364.62
Apr 3	335.29	-17.65	160.60	-147.69
Apr 4	273.53	-79.41	270.16	67.69
Apr 5	276.47	-76.47	341.11	126.15

Dry Runs	Turning distance from base (mm)	Turning height from wall (mm)	Block Height from wall (mm)	Block Dist from centre (mm)
Mar 2 Trial 1	400.00	47.06	41.18	303.67
Mar 2 Trial 2	382.35	29.41	47.06	287.27
Mar 2 Trial 3	282.35	-70.59	47.06	275.72
Mar 2 Trial 4	276.47	-76.47	29.41	156.96
Mar 5 Trial 1	300.00	-52.94	158.82	228.81
Mar 5 Trial 2	394.12	41.18	47.06	313.33
Apr 5 Trial 2	411.76	58.82	47.06	272.83
Aug 6 Trial 1	402.94	50.00	58.82	370.40

No Fan Runs	NF Turning Distance from base (mm)	NF Turning height from wall (mm)	NF Block distance from centre (mm)	NF Block height from wall (mm)
Mar28 Trial 1	332.94	-20.00	356.88	56.92
Mar28 Trial 2	373.53	20.59	437.38	20.00
Apr4 Trial2	452.94	100.00	312.38	304.62
Aug 8 Trial1	550.00	197.06	422.88	49.23
Nov 17 Trial 1	383.71	30.77	246.43	-127.69
Nov 17 Trial 3	405.25	52.31	256.11	-209.23
Nov 18 Trial 1	383.71	30.77	273.64	-144.62
Dec 6 Trial 1	443.71	90.77	522.90	233.85
Dec 17 Trial 1	380.63	27.69	383.66	218.46

Fan Runs	Fan Turning distance from base (mm)	Fan Turning distance from wall (mm)	Fan Block distance from centre (mm)	Fan Block height (mm)
Mar 29	332.35	-20.59	321.94	64.62
Mar 30	347.06	-5.88	660.19	375.38
Apr 2	314.71	-38.24	315.14	280.00
Apr 3	350.00	-2.94	393.30	192.31
Apr 4	338.24	-14.71	218.55	126.15
Apr 5	302.94	-50.00	151.52	33.85

Dry Runs	*Turning distance from base (mm)*	*Turning height from wall (mm)*	*Block Height from wall (mm)*	*Block Dist from centre (mm)*
Mar 2 Trial 1	364.71	11.76	17.65	254.03
Mar 2 Trial 2	364.71	11.76	70.59	160.56
Mar 2 Trial 3	352.94	0.00	182.35	287.75
Mar 2 Trial 4	300.00	-52.94	170.59	192.33
Mar 5 Trial 1	352.94	0.00	58.82	185.20
Mar 5 Trial 2	373.53	20.59	167.65	269.26
Apr 5 Trial 2	426.47	73.53	50.00	273.42
Aug 6 Trial 1	379.41	8.82	185.29	328.91

No Fan Runs	*NF Turning Distance from base (mm)*	*NF Turning height from wall (mm)*	*NF Block distance from centre (mm)*	*NF Block height from wall (mm)*
Mar28 Trial 1	560.63	207.69	332.33	146.15
Mar28 Trial 2	438.24	85.29	230.19	33.85
Apr4 Trial2	400.00	47.06	356.33	70.77
Aug 8 Trial1	700.00	347.06	658.32	424.62
Nov 17 Trial 1	369.86	16.92	248.00	190.77
Nov 17 Trial 3	569.86	216.92	234.14	-29.23
Nov 18 Trial 1	440.63	87.69	367.92	49.23
Dec 6 Trial 1	508.32	155.38	354.13	141.54
Dec 17 Trial 1	531.4	178.46	315.70	46.15

Fan Runs	*Fan Turning distance from base (mm)*	*Fan Turning distance from wall (mm)*	*Fan Block distance from centre (mm)*	*Fan Block height (mm)*
Mar 29	367.65	14.71	500.15	246.15
Mar 30	329.41	-23.53	317.91	40.00
Apr 2	361.76	8.82	276.12	273.85
Apr 3	300.00	-52.94	33.03	29.23
Apr 4	355.88	2.94	265.21	260.00
Apr 5	341.18	-11.76	302.49	49.23

Dry Runs	Turning distance from base (mm)	Turning height from wall (mm)	Block Height from wall (mm)	Block Dist from centre (mm)
Mar 2 Trial 1	376.47	23.53	35.29	227.29
Mar 2 Trial 2	400.00	47.06	58.82	129.14
Mar 2 Trial 3	294.12	-58.82	-58.82	235.44
Mar 2 Trial 4	305.88	-47.06	111.76	137.20
Mar 5 Trial 1	305.88	-47.06	23.53	41.59
Mar 5 Trial 2	300.00	-52.94	82.35	245.80
Apr 5 Trial 2	367.65	14.71	126.47	349.55
Aug 6 Trial 1	361.76	8.82	164.71	407.97

No Fan Runs	NF Turning Distance from base (mm)	NF Turning height from wall (mm)	NF Block distance from centre (mm)	NF Block height from wall (mm)
Mar28 Trial 1	352.94	0.00	408.25	195.38
Mar28 Trial 2	497.06	144.12	366.00	46.15
Apr4 Trial2	647.06	294.12	216.62	147.69
Aug 8 Trial1	552.94	200.00	474.31	69.23
Nov 17 Trial 1	432.94	80.00	438.54	126.15
Nov 17 Trial 3	560.63	207.69	457.73	126.15
Nov 18 Trial 1	369.86	16.92	596.56	240.00
Dec 6 Trial 1	405.25	52.31	376.50	364.62
Dec 17 Trial 1	385.25	32.31	243.30	72.31

Fan Runs	Fan Turning distance from base (mm)	Fan Turning distance from wall (mm)	Fan Block distance from centre (mm)	Fan Block height (mm)
Mar 29	332.35	-20.59	424.09	203.08
Mar 30	341.18	-11.76	158.58	-6.15
Apr 2	370.59	17.65	348.97	120.00
Apr 3	300.00	-52.94	389.46	276.92
Apr 4	323.53	-29.41	278.81	278.46
Apr 5	314.71	-38.24	89.51	43.08

Dry Runs	*Turning distance from base (mm)*	*Turning height from wall (mm)*	*Block Height from wall (mm)*	*Block Dist from centre (mm)*
Mar 2 Trial 1	429.41	76.47	217.65	274.21
Mar 2 Trial 2	405.88	52.94	123.53	228.43
Mar 2 Trial 3	288.24	-64.71	-47.06	158.39
Mar 2 Trial 4	270.59	-82.35	-76.47	234.41
Mar 5 Trial 1	388.24	35.29	23.53	248.80
Mar 5 Trial 2	308.82	-44.12	47.06	304.64
Apr 5 Trial 2	400.00	47.06	202.94	325.74
Aug 6 Trial 1	397.06	44.12	120.59	214.73

No Fan Runs	*NF Turning Distance from base (mm)*	*NF Turning height from wall (mm)*	*NF Block distance from centre (mm)*	*NF Block height from wall (mm)*
Mar28 Trial 1	529.86	176.92	545.48	544.62
Mar28 Trial 2	479.41	126.47	404.78	126.15
Apr4 Trial2	567.65	214.71	629.95	583.08
Aug 8 Trial1	497.06	144.12	565.03	224.62
Nov 17 Trial 1	351.4	-1.54	274.67	46.15
Nov 17 Trial 3	562.17	209.23	379.86	75.38
Nov 18 Trial 1	416.02	63.08	521.09	195.38
Dec 6 Trial 1	549.86	196.92	521.37	510.77
Dec 17 Trial 1	359.09	6.15	387.15	-27.69

Fan Runs	*Fan Turning distance from base (mm)*	*Fan Turning distance from wall (mm)*	*Fan Block distance from centre (mm)*	*Fan Block height (mm)*
Mar 29	373.53	20.59	188.92	21.54
Mar 30	379.41	26.47	592.76	336.92
Apr 2	420.59	67.65	317.82	109.23
Apr 3	323.53	-29.41	235.51	-7.69
Apr 4	364.71	11.76	102.42	86.15
Apr 5	355.88	2.94	232.31	0.00

Dry Runs	*Turning distance from base (mm)*	*Turning height from wall (mm)*	*Block Height from wall (mm)*	*Block Dist from centre (mm)*
Mar 2 Trial 1	352.94	0.00	158.82	224.76
Mar 2 Trial 2	305.88	-47.06	135.29	176.47
Mar 2 Trial 3	282.35	-70.59	-52.94	216.93
Mar 2 Trial 4	258.82	-94.12	-52.94	154.40
Mar 5 Trial 1	282.35	-70.59	117.65	155.30
Mar 5 Trial 2	270.59	-82.35	-76.47	242.84
Apr 5 Trial 2	397.06	44.12	150.00	258.24
Aug 6 Trial 1	373.53	20.59	147.06	349.90

No Fan Runs	*NF Turning Distance from base (mm)*	*NF Turning height from wall (mm)*	*NF Block distance from centre (mm)*	*NF Block height from wall (mm)*
Mar28 Trial 1	532.71	179.77	485.89	296.92
Mar28 Trial 2	508.82	155.88	220.88	70.77
Apr4 Trial2	538.24	185.29	564.80	524.62
Aug 8 Trial1	667.65	314.71	470.54	140.00
Nov 17 Trial 1	423.71	70.77	531.48	404.62
Nov 17 Trial 3	420.63	67.69	574.39	406.15
Nov 18 Trial 1	383.71	30.77	454.73	181.54
Dec 6 Trial 1	403.71	50.77	406.17	110.77
Dec 17 Trial 1	397.56	44.62	306.32	156.92

Fan Runs	*Fan Turning distance from base (mm)*	*Fan Turning distance from wall (mm)*	*Fan Block distance from centre (mm)*	*Fan Block height (mm)*
Mar 29	326.47	-26.47	353.88	187.69
Mar 30	258.82	-94.12	223.16	6.15
Apr 2	358.82	5.88	242.89	46.15
Apr 3	335.29	-17.65	248.38	244.62
Apr 4	332.35	-20.59	160.01	116.92
Apr 5	361.76	8.82	393.87	240.00

Dry Runs	Turning distance from base (mm)	Turning height from wall (mm)	Block Height from wall (mm)	Block Dist from centre (mm)
Mar 2 Trial 1	370.59	17.65	70.59	225.15
Mar 2 Trial 2	288.24	-64.71	23.53	231.29
Mar 2 Trial 3	417.65	64.71	129.41	236.61
Mar 2 Trial 4	311.76	-41.18	29.41	232.11
Mar 5 Trial 1	352.94	0.00	47.06	53.27
Mar 5 Trial 2	355.88	2.94	-2.94	182.38
Apr 5 Trial 2	382.35	29.41	100.00	149.74
Aug 6 Trial 1	350.00	-2.94	85.29	274.41

No Fan Runs	NF Turning Distance from base (mm)	NF Turning height from wall (mm)	NF Block distance from centre (mm)	NF Block height from wall (mm)
Mar28 Trial 1	757.56	404.62	469.24	469.23
Mar28 Trial 2	523.53	170.59	357.43	173.85
Apr4 Trial2	552.94	200.00	236.19	215.38
Aug 8 Trial1	520.59	167.65	339.21	50.77
Nov 17 Trial 1	534.48	181.54	370.32	175.38
Nov 17 Trial 3	594.48	241.54	245.81	-118.46
Nov 18 Trial 1	525.25	172.31	374.29	333.85
Dec 6 Trial 1	463.71	110.77	338.06	83.08
Dec 17 Trial 1	460.79	107.85	293.92	215.38

Fan Runs	Fan Turning distance from base (mm)	Fan Turning distance from wall (mm)	Fan Block distance from centre (mm)	Fan Block height (mm)
Mar 29	332.35	-20.59	171.41	150.77
Mar 30	300.00	-52.94	95.95	63.08
Apr 2	352.94	0.00	159.15	46.15
Apr 3	367.65	14.71	492.50	463.08
Apr 4	320.59	-32.35	62.79	32.31
Apr 5	373.53	20.59	324.93	196.92

Dry Runs	Turning distance from base (mm)	Turning height from wall (mm)	Block Height from wall (mm)	Block Dist from centre (mm)
Mar 2 Trial 1	405.88	52.94	70.59	197.74
Mar 2 Trial 2	247.06	-105.88	-52.94	199.74
Mar 2 Trial 3	300.00	-52.94	23.53	104.24
Mar 2 Trial 4	311.76	-41.18	52.94	83.19
Mar 5 Trial 1	305.88	-47.06	17.65	160.56
Mar 5 Trial 2	370.59	17.65	76.47	229.96
Apr 5 Trial 2	414.71	61.76	-29.41	134.43
Aug 6 Trial 1	367.65	14.71	147.06	160.02

No Fan Runs	NF Turning Distance from base (mm)	NF Turning height from wall (mm)	NF Block distance from centre (mm)	NF Block height from wall (mm)
Mar28 Trial 1	757.56	404.62	521.09	195.38
Mar28 Trial 2	629.41	276.47	520.18	120.00
Apr4 Trial2	591.18	238.24	398.50	396.92
Aug 8 Trial1	700.00	347.06	271.14	126.15
Nov 17 Trial 1	577.56	224.62	386.53	200.00
Nov 17 Trial 3	622.17	269.23	201.87	50.77
Nov 18 Trial 1	471.4	118.46	396.32	240.00
Dec 6 Trial 1	588.32	235.38	488.17	266.15
Dec 17 Trial 1	443.71	90.77	325.75	81.54

Fan Runs	Fan Turning distance from base (mm)	Fan Turning distance from wall (mm)	Fan Block distance from centre (mm)	Fan Block height (mm)
Mar 29	335.29	-17.65	244.32	-12.31
Mar 30	347.06	-5.88	253.47	-12.31
Apr 2	314.71	-38.24	154.13	126.15
Apr 3	370.59	17.65	153.63	113.85
Apr 4	250.00	-102.94	123.08	73.85
Apr 5	323.53	-29.41	242.89	16.92

Dry Runs	*Turning distance from base (mm)*	*Turning height from wall (mm)*	*Block Height from wall (mm)*	*Block Dist from centre (mm)*
Mar 2 Trial 1	276.47	-76.47	-35.29	196.33
Mar 2 Trial 2	358.82	5.88	52.94	121.13
Mar 2 Trial 3	335.29	-17.65	0.00	123.67
Mar 2 Trial 4	258.82	-94.12	-41.18	203.09
Mar 5 Trial 1	264.71	-88.24	-41.18	42.42
Mar 5 Trial 2	302.94	-50.00	58.82	160.66
Apr 5 Trial 2	335.29	-17.65	91.18	99.87
Aug 6 Trial 1	402.94	50.00	226.47	235.97

No Fan Runs	*NF Turning Distance from base (mm)*	*NF Turning height from wall (mm)*	*NF Block distance from centre (mm)*	*NF Block height from wall (mm)*
Mar28 Trial 1	948.32	595.38	616.59	338.46
Mar28 Trial 2	597.06	244.12	439.34	143.08
Apr4 Trial2	511.76	158.82	238.86	13.85
Aug 8 Trial1	720.59	367.65	544.85	469.23
Nov 17 Trial 1	432.94	80.00	219.53	93.85
Nov 17 Trial 3	568.32	215.38	598.23	593.85
Nov 18 Trial 1	446.79	93.85	382.26	144.62
Dec 6 Trial 1	528.32	175.38	400.64	190.77
Dec 17 Trial 1	520.63	167.69	344.48	332.31

Fan Runs	*Fan Turning distance from base (mm)*	*Fan Turning distance from wall (mm)*	*Fan Block distance from centre (mm)*	*Fan Block height (mm)*
Mar 29	341.18	-11.76	221.58	95.38
Mar 30	376.47	23.53	168.65	116.92
Apr 2	382.35	29.41	111.97	-24.62
Apr 3	350.00	-2.94	317.82	298.46
Apr 4	270.59	-82.35	36.05	-23.08
Apr 5	364.71	11.76	110.94	61.54

Dry Runs	Turning distance from base (mm)	Turning height from wall (mm)	Block Height from wall (mm)	Block Dist from centre (mm)
Mar 2 Trial 1	352.94	0.00	0.00	188.33
Mar 2 Trial 2	294.12	-58.82	-58.82	218.28
Mar 2 Trial 3	276.47	-76.47	-47.06	58.23
Mar 2 Trial 4	288.24	-64.71	-17.65	21.21
Mar 5 Trial 1				
Mar 5 Trial 2	350.00	-2.94	208.82	219.19
Apr 5 Trial 2	367.65	14.71	52.94	77.20
Aug 6 Trial 1	358.82	5.88	150.00	212.30

No Fan Runs	NF Turning Distance from base (mm)	NF Turning height from wall (mm)	NF Block distance from centre (mm)	NF Block height from wall (mm)
Mar28 Trial 1	777.56	424.62	528.68	441.54
Mar28 Trial 2	608.82	255.88	246.79	150.77
Apr4 Trial2	544.12	191.18	334.44	333.85
Aug 8 Trial1	700.00	347.06	553.65	293.85
Nov 17 Trial 1	794.48	441.54	371.15	253.85
Nov 17 Trial 3	479.09	126.15	454.11	260.00
Nov 18 Trial 1	439.09	86.15	312.31	121.54
Dec 6 Trial 1	560.63	207.69	477.60	333.85
Dec 17 Trial 1	579.09	226.15	426.64	364.62

Fan Runs	Fan Turning distance from base (mm)	Fan Turning distance from wall (mm)	Fan Block distance from centre (mm)	Fan Block height (mm)
Mar 29	285.29	-67.65	275.38	267.69
Mar 30	317.65	-35.29	16.06	15.38
Apr 2	347.06	-5.88	172.32	170.77
Apr 3	332.35	-20.59	125.00	-29.23
Apr 4	273.53	-79.41	151.40	-13.85
Apr 5	270.59	-82.35	221.37	67.69

Dry Runs	Turning distance from base (mm)	Turning height from wall (mm)	Block Height from wall (mm)	Block Dist from centre (mm)
Mar 2 Trial 1	376.47	23.53	70.59	145.28
Mar 2 Trial 2	352.94	0.00	0.00	35.78
Mar 2 Trial 3	235.29	-117.65	-64.71	79.14
Mar 2 Trial 4	282.35	-70.59	-41.18	106.05
Mar 5 Trial 1				
Mar 5 Trial 2	414.71	61.76	108.82	191.18
Apr 5 Trial 2				
Aug 6 Trial 1	367.65	14.71	135.29	144.84

No Fan Runs	NF Turning Distance from base (mm)	NF Turning height from wall (mm)	NF Block distance from centre (mm)	NF Block height from wall (mm)
Mar28 Trial 1	717.56	364.62	733.23	521.54
Mar28 Trial 2	711.76	358.82	329.67	236.92
Apr4 Trial2	602.94	250.00	500.62	475.38
Aug 8 Trial1	650.00	297.06	596.16	424.62
Nov 17 Trial 1	996.02	643.08	598.45	589.23
Nov 17 Trial 3	483.71	130.77	375.26	352.31
Nov 18 Trial 1	456.02	103.08	231.99	96.92
Dec 6 Trial 1	588.32	235.38	361.85	215.38
Dec 17 Trial 1	525.25	172.31	370.46	296.92

Fan Runs	Fan Turning distance from base (mm)	Fan Turning distance from wall (mm)	Fan Block distance from centre (mm)	Fan Block height (mm)
Mar 29	300.00	-52.94	126.91	-64.62
Mar 30				
Apr 2	326.47	-26.47	105.57	100.00
Apr 3	305.88	-47.06	179.64	158.46
Apr 4	250.00	-102.94	78.45	-52.31
Apr 5	247.06	-105.88	59.19	-27.69

Dry Runs	*Turning distance from base (mm)*	*Turning height from wall (mm)*	*Block Height from wall (mm)*	*Block Dist from centre (mm)*
Mar 2 Trial 1	364.71	11.76	41.18	126.71
Mar 2 Trial 2				
Mar 2 Trial 3				
Mar 2 Trial 4	276.47	-76.47	-82.35	80.01
Mar 5 Trial 1				
Mar 5 Trial 2	258.82	-94.12	123.53	151.83
Apr 5 Trial 2				
Aug 6 Trial 1	385.29	32.35	41.18	194.03

No Fan Runs	*NF Turning Distance from base (mm)*	*NF Turning height from wall (mm)*	*NF Block distance from centre (mm)*	*NF Block height from wall (mm)*
Mar28 Trial 1	706.79	353.85	657.07	463.08
Mar28 Trial 2	608.82	255.88	550.07	215.38
Apr4 Trial2	638.24	285.29	430.30	396.92
Aug 8 Trial1	652.94	300.00	506.27	312.31
Nov 17 Trial 1	769.86	416.92	593.11	527.69
Nov 17 Trial 3	549.86	196.92	557.69	463.08
Nov 18 Trial 1	631.4	278.46	286.27	195.38
Dec 6 Trial 1	571.4	218.46	347.41	233.85
Dec 17 Trial 1	597.56	244.62	259.18	238.46

Fan Runs	*Fan Turning distance from base (mm)*	*Fan Turning distance from wall (mm)*	*Fan Block distance from centre (mm)*	*Fan Block height (mm)*
Mar 29	323.53	-29.41	16.06	-4.62
Mar 30				
Apr 2	305.88	-47.06	101.74	35.38
Apr 3	335.29	-17.65	58.97	56.92
Apr 4	244.12	-108.82	150.69	-96.92
Apr 5	258.82	-94.12	154.61	-104.62

Dry Runs	*Turning distance from base (mm)*	*Turning height from wall (mm)*	*Block Height from wall (mm)*	*Block Dist from centre (mm)*
Mar 2 Trial 1	352.94	0.00	129.41	145.28
Mar 2 Trial 2				
Mar 2 Trial 3				
Mar 2 Trial 4				
Mar 5 Trial 1				
Mar 5 Trial 2	302.94	-50.00	100.00	105.92
Apr 5 Trial 2				
Aug 6 Trial 1	444.12	91.18	120.59	267.13

No Fan Runs	*NF Turning Distance from base (mm)*	*NF Turning height from wall (mm)*	*NF Block distance from centre (mm)*	*NF Block height from wall (mm)*
Mar28 Trial 1	736.02	383.08	570.54	386.15
Mar28 Trial 2	750.00	397.06	357.99	333.85
Apr4 Trial2	714.71	361.76	405.54	389.23
Aug 8 Trial1	720.59	367.65	471.98	396.92
Nov 17 Trial 1	794.48	441.54	497.09	424.62
Nov 17 Trial 3	568.32	215.38	525.32	518.46
Nov 18 Trial 1	646.79	293.85	287.80	30.77
Dec 6 Trial 1	479.09	126.15	248.64	52.31
Dec 17 Trial 1	602.17	249.23	300.28	215.38

Fan Runs	*Fan Turning distance from base (mm)*	*Fan Turning distance from wall (mm)*	*Fan Block distance from centre (mm)*	*Fan Block height (mm)*
Mar 29				
Mar 30				
Apr 2				
Apr 3	4.22	30.77	-1.54	
Apr 4				
Apr 5				

Dry (distance)	Dry (angle)	Fan (distance)	Fan (angle)		Min distance Dry	Min Distance Fan		Dry (height)	Fan (height)
379.7649974	16.18920626	241.54	65.81		151.8342294	35.38		100	0
280.4469986	9.659893078	275.22	50.67		51.36543881	159.60		41.17647059	-152.3076923
325.6614244	16.79837178	227.20	60.26		109.4576122	68.96		88.23529412	-43.07692308
311.0980664	164.6498635	333.12	-42.15		89.59733066	75.13		76.47058824	50.76923077
293.0569108	169.5922887	337.66	-53.02		57.93445766	113.58		47.05882353	103.0769231
295.5845771	174.2894069	194.66	55.30		41.59451654	114.82		23.52941176	-66.15384615
170.5882353	180	234.98	-5.52		94.11764706	89.61455926		-5.882352941	-73.84615385
307.0255785	4.945966547	236.51	63.03		56.574659	47.51829296		20.58823529	-23.07692308
326.7222136	13.53585637	384.83	156.23		98.30	394.5576506		70.58823529	364.6153846
249.7057091	43.09084757	160.60	37.03		185.76	259.8907372		164.7058824	-147.6923077
263.0668209	169.6951535	270.16	-26.57		47.43	68.80209162		41.17647059	67.69230769
295.5845771	148.8406955	341.11	-59.47		153.39	133.3057171		147.0588235	126.1538462
286.8514198	151.8583988	321.9357134	-41.83963837		135.80	76.81530913		129.4117647	64.61538462
372.7991936	9.536635758	660.1864538	-117.2736201		127.70	461.9510976		55.88235294	375.3846154
209.4026766	-10.52078431	315.1406186	147.8042661		62.94	308.3838383		-44.11764706	280
340.8213162	21.25050551	393.2988986	-88.19126068		138.11	204.3896962		117.6470588	192.3076923
303.6684588	171.085073	218.5481952	14.62087399		58.82352941	158.1550434		41.17647059	126.1538462
287.2733046	169.3803447	151.5208893	64.6758186		55.80489989	133.5894975		47.05882353	33.84615385
275.7186269	11.07020258	500.1514564	-101.6649204		54.94571086	294.4255263		47.05882353	246.1538462
156.9607537	12.99461679	317.9110813	-37.04922657		108.8235294	57.66666097		29.41176471	40
228.807651	133.9583733	276.1227752	115.4633451		195.8037291	363.1192935		158.8235294	273.8461538
313.3284866	170.2724214	33.03217008	47.27044967		68.91396773	260.1092169		47.05882353	29.23076923
272.8327624	168.8112001	265.2095298	104.2025588		53.02281287	343.5870647		47.05882353	260
370.4014469	169.9393102	302.4945596	-34.50852299		119.108569	55.04167329		58.82352941	49.23076923
254.0332104	5.31454567	424.0910921	-92.00955381		23.71	225.6876409		17.64705882	203.0769231
160.556989	28.44292862	158.5809856	60.2551187		137.86	118.6212709		70.58823529	-6.153846154
287.7547014	40.85537626	348.9731344	81.65830156		192.08	130.2977927		182.3529412	120
192.3268556	66.57130719	389.4587353	-136.1233027		251.66	276.9230769		170.5882353	276.9230769
185.2007234	159.5504524	278.8055673	114.0071466		111.80	380.9735272		58.82352941	278.4615385
269.2582404	139.8738964	89.50885051	60.80968848		183.23	203.0827505		167.6470588	43.07692308
273.4186999	11.79342968	188.9240792	3.012787504		57.11	88.80535194		50	21.53846154
328.9124367	35.53767779	592.7570723	-115.7099538		191.54	399.0580625		185.2941176	336.9230769
227.2903422	10.43747535	317.8217282	-49.18491613		52.37	111.9699875		35.29411765	109.2307692
129.1441082	30.06858282	235.5102731	-10.64566334		157.98	39.22322703		58.82352941	-7.692307692

235.4411305	-167.0053832	102.4204122	51.95295747		63.63	234.8359619		-58.82352941	86.15384615
137.1988681	59.03624347	232.3076923	-10.18662976		219.49	41.53846154		111.7647059	0
41.59451654	135	353.8762529	-84.88268515		237.13	189.51		23.52941176	187.6923077
245.7950872	158.962489	223.1617876	-7.806400829		95.03	51.14		82.35294118	6.153846154
349.5548331	22.24902366	242.8869754	-15.40342496		148.64	58.16		126.4705882	46.15384615
407.9656679	155.2825591	248.3793625	107.2234362		200.78	336.29		164.7058824	244.6153846
274.2085181	54.60520416	160.0073963	75.35568736		243.6920457	204.43		217.6470588	116.9230769
228.4292687	145.491477	393.8731962	-110.4722795		150.316851	243.06		123.5294118	240
158.3872002	-164.9315118	171.4056057	80.58901808		119.108569	246.7924409		-47.05882353	150.7692308
234.4101041	-17.52556837	95.95363772	63.56606022		77.64928696	214.1171498		-76.47058824	63.07692308
248.8033645	6.788974574	159.1471352	16.48445319		30.70678385	130.0068273		23.52941176	46.15384615
304.6354677	10.0079798	492.5047682	173.7325186		68.91396773	475.088282		47.05882353	463.0769231
325.7411034	39.87180896	62.79486656	56.7174347		208.906363	225.4043047		202.9411765	32.30769231
214.7260265	143.914927	324.9324008	-90		155.9100974	197.5231243		120.5882353	196.9230769
224.7643903	47.1210964	244.3200927	-9.865806943		194.2290233	141.7389884		158.8235294	-12.30769231
176.4705882	126.8698976	253.4729191	-12.87500156		212.4987279	156.9909356		135.2941176	-12.30769231
216.9304578	-12.52880771	154.1305064	64.56378362		64.50503588	244.9248164		-52.94117647	126.1538462
154.4047617	-17.74467163	153.6306183	59.2645123		118.5626139	230.4202489		-52.94117647	113.8461538
155.2985739	127.3039483	123.0769231	20.55604522		210.6178093	150.7692308		117.6470588	73.84615385
242.8386053	-16.89864869	242.8869754	26.56505118		74.40653318	188.1520221		-76.47058824	16.92307692
258.237974	37.13092445	221.5811925	75.65066796		163.7049418	122.5372785		150	95.38461538
349.901123	154.0811144	168.6493509	43.29864539		160.9068161	192.0120805		147.0588235	116.9230769
225.1489319	160.1447856	111.9699875	21.59531045		93.00816648	166.4456128		70.58823529	-24.61538462
231.28945	172.6942405	317.8217282	139.1849161		45.94264515	340.8482284		23.52941176	298.4615385
236.6139454	34.87532834	36.0473062	37.56859203		148.7258456	247.2332105		129.4117647	-23.07692308
232.1108195	171.2538377	110.9400392	37.69424047		49.91341985	191.6851462		29.41176471	61.53846154
53.2669714	96.34019175	275.380318	105.1409838		264.1824891	341.6631789		47.05882353	267.6923077
182.3766588	0.9240453528	16.06201001	46.6500394		73.58821178	269.6699714		-2.941176471	15.38461538
149.7402595	45	172.321428	83.22043205		183.6057534	305.9411708		100	170.7692308
274.4134985	160.5940292	125.0041419	18.43494882		91.36602686	155.0873012		85.29411765	-29.23076923
197.7380743	22.75097634	151.4036886	58.7759789		106.0864047	126.9114214		70.58823529	-13.84615385
199.7403158	-166.3730051	221.3728013	-6.115503566		84.8365006	92.52538433		-52.94117647	67.69230769
104.2355597	163.6104597	126.9114214	18.53599948		167.3113253	176.8427911		23.52941176	-64.61538462
83.18903308	45	105.5725444	67.21759427		205.6511307	262.8429009		52.94117647	100

160.556989	8.426969021	179.6380318	82.22086281		99.87015792	249.1832813		17.64705882	158.4615385
229.9578719	20.98489784	78.44645406	45.69869438		92.07338731	224.6364584		76.47058824	-52.30769231
134.4281751	-10.08059799	59.18579711	32.77487732		125.7503431	223.2625223		-29.41176471	-27.69230769
160.0173001	72.89727103	16.06201001	42.01136754		258.8402401	258.50		147.0588235	-4.615384615
196.3331679	-8.615648184	101.7364038	61.07357416		68.4100197	184.95		-35.29411765	35.38461538
121.1250605	150.9453959	58.96543968	52.36107767		169.3668241	264.66		52.94117647	56.92307692
123.6693885	2.726310994	150.6907174	15.5086382		132.4835956	185.75		0	-96.92307692
203.0903118	-169.9920202	154.6134711	15.40342496		73.70567109	191.17		-41.17647059	-104.6153846
42.4182503	-56.30993247	33.88			235.0182206			-41.17647059	-1.54
160.6647092	23.74949449				126.6073132			58.82352941	
99.87015792	76.37300514				251.8100564			91.17647059	
235.9732845	79.95065141				316.3645132			226.4705882	
188.3271835	178.2100894				76.69649888			0	
218.2820625	-14.03624347				68.91396773			-58.82352941	
58.23232316	-135				227.2903422			-47.05882353	
21.20912515	-33.69006753				238.5256038			-17.64705882	
219.1916575	78.38851358				301.5674163			208.8235294	
77.20238087	130.3645366				222.6181114			52.94117647	
212.2950874	47.24574257				191.8089087			150	
145.2834004	31.75948008				152.8563113			70.59	
35.78095606	170.5376778				229.4871671			0.00	
79.13896498	-48.0127875				211.2944124			-64.71	
106.0456257	-160.5599652				168.4449537			-41.18	
191.1764706	143.1301024				160.1523928			108.82	
144.8361441	102.9074087				271.8799094			135.29	
126.7097602	158.1985905				154.40			41.17647059	
80.00865005	-107.102729				253.01			-82.35294118	
151.8342294	58.46520809				218.84			123.5294118	
194.0285	165.9637565				89.79			41.17647059	
145.2834004	111.3706223				251.2942278			129.4117647	
105.9231947	88.40885973				274.2085181			100	
267.1294283	28.25802961				128.1354171			120.5882353	
